# The Danger Signal S100B Integrates Pathogen– and Danger–Sensing Pathways to Restrain Inflammation

**DOI:** 10.1371/journal.ppat.1001315

**Published:** 2011-03-10

**Authors:** Guglielmo Sorci, Gloria Giovannini, Francesca Riuzzi, Pierluigi Bonifazi, Teresa Zelante, Silvia Zagarella, Francesco Bistoni, Rosario Donato, Luigina Romani

**Affiliations:** Department of Experimental Medicine and Biochemical Sciences, University of Perugia, Perugia, Italy; University of Massachusetts Medical School, United States of America

## Abstract

Humans inhale hundreds of *Aspergillus* conidia without adverse consequences. Powerful protective mechanisms may ensure prompt control of the pathogen and inflammation. Here we reveal a previously unknown mechanism by which the danger molecule S100B integrates pathogen– and danger–sensing pathways to restrain inflammation. Upon forming complexes with TLR2 ligands, S100B inhibited TLR2 via RAGE, through a paracrine epithelial cells/neutrophil circuit that restrained pathogen-induced inflammation. However, upon binding to nucleic acids, S100B activated intracellular TLRs eventually resolve danger-induced inflammation via transcriptional inhibition of S100B. Thus, the spatiotemporal regulation of TLRs and RAGE by S100B provides evidence for an evolving braking circuit in infection whereby an endogenous danger protects against pathogen–induced inflammation and a pathogen–sensing mechanism resolves danger–induced inflammation.

## Author Summary

Inflammation results from recognition of invading microorganisms through pathogen–associated molecular patterns (PAMPs) and from reaction to tissue damage–associated molecular patterns (DAMPs). Despite the identification of specific signaling pathways negatively regulating responses to PAMPs or DAMPs, the unexpected convergence of molecular pathways responsible for recognition of either one raised the question of whether and how the host discriminates between the two distinct molecular patterns. Here we reveal a previously unknown mechanism by which the danger molecule S100B integrates pathogen– and danger–sensing pathways to restrain inflammation in *Aspergillus fumigatus* infection. By disclosing protective mechanisms that ensure prompt control of the pathogen and inflammation, our results may help to explain why humans inhale hundreds of *Aspergillus* conidia without adverse consequences.

## Introduction

Inflammation results from recognition of invading microorganisms through pathogen–associated molecular patterns (PAMPs) and from reaction to tissue damage–associated molecular patterns (DAMPs) [1], [2]. It is known that the innate immune system recognizes both PAMPs and DAMPs through pattern recognition receptors, such as Toll–like receptors (TLRs) and other receptors [3], [4], [5], [6]. Multiple positive feedback loops between DAMPs and PAMPs and their overlapping receptors temporally and spatially drive immune regulatory functions. Despite the identification of specific signaling pathways negatively regulating responses to PAMPs or DAMPs [7], [8], the unexpected convergence of molecular pathways responsible for recognition of PAMPs and DAMPs raised the question of whether and how the host discriminates between these two molecular patterns [9], [10].

DAMPs such as the high mobility group box 1 protein (HMGB1) and S100 proteins represent important danger signals that, although primarily intracellular, may mediate inflammatory responses through autocrine/paracrine interactions with the receptor for advanced glycation end–products (RAGE), a multiligand receptor of the immunoglobulin superfamily [3], [4], [5], [11], [12]. Integral to the biology of RAGE and its ligands is their up–regulation and increased accumulation in multiple biological and disease settings. The ability to activate expression programs that encode innate immune responsive genes confers to RAGE a central role in chronic inflammatory diseases.

Engagement of RAGE converts a brief pulse of cellular activation to sustained cellular dysfunction, eventually leading to inflammation [4] and tumor promotion [13]. However, because RAGE is expressed in multiple, distinct cell types, including immune cells, and both murine and human RAGE genes undergo extensive splicing with distinct splice isoforms being uniquely distributed in different tissues [14], it is not surprising that diverse signal transduction and effector pathways may be impacted by RAGE depending on sites, ligands and time course of ligand–RAGE stimulation [15], [16], [17]. The complexity of the system is enhanced by the findings that the ligands of RAGE may interact with distinct TLR–binding molecules thus amplifying inflammatory and immune responses in infection [11], [18], [19], [20]. Thus, although promoting pathology, RAGE signaling also contributes to beneficial, inflammatory mechanisms of repair, in certain settings [5]. Ultimately, discerning the primal versus the chronic injury–provoking roles for this ligand–receptor interaction is a challenge in delineating the functions of the ligand/RAGE axis [21].

Given that RAGE is expressed at the highest levels in the lung compared to other tissues [5], [22] and both protects and causes lung injury [5], the DAMP/RAGE axis likely integrates with the PAMP/TLR axis in the inflammatory responses in lung infections. We have addressed whether and how the two systems interact in a mouse model of pulmonary infection with a model fungal pathogen as well a common cause of severe infections and diseases, *Aspergillus fumigatus* [23]. Humans inhale hundreds of conidia per day without adverse consequences [24], except for a small minority of persons in whom defense systems fail and a life–threatening angioinvasive form of aspergillosis can develop. Some degree of inflammation is required for protection during the transitional response occurring between the rapid innate and slower adaptive response. However, progressive inflammation worsens disease and ultimately prevents pathogen eradication, a condition in which it is an exaggerated inflammatory response that likely compromises a host's ability to eradicate infection and not an “intrinsic” susceptibility to infection that determines a state of chronic or intractable disease [25]. We disclosed the complexity of signalling integration between different innate immune biosensors by showing that the spatiotemporal regulation of TLRs and RAGE by S100B limits pathogen– as well as danger–induced inflammation and ensures protection in infection.

## Results

### RAGE and DAMPs expression in pulmonary aspergillosis

**Figure 1:**
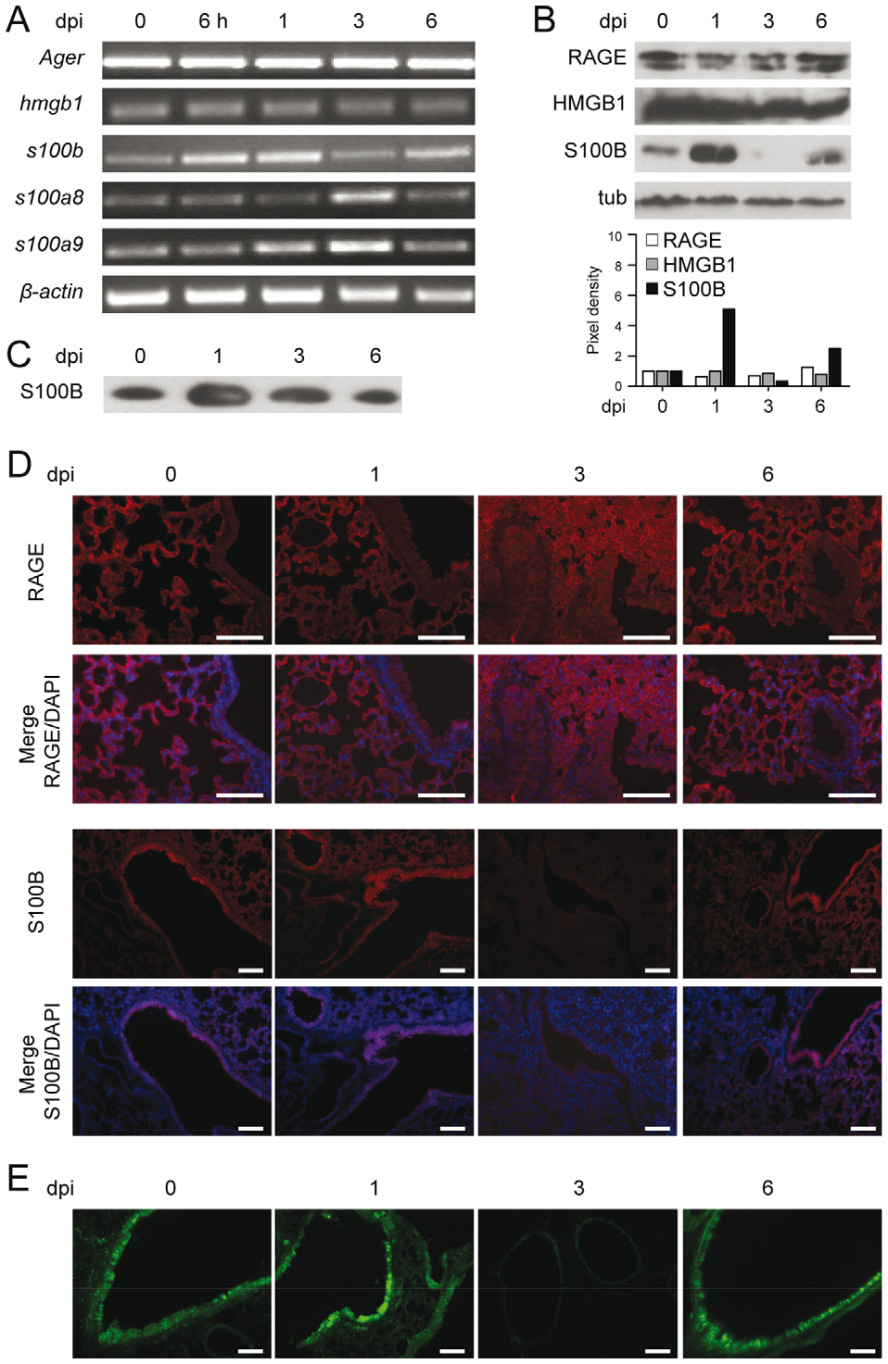
RAGE and DAMPs expression in pulmonary aspergillosis. Expression of RAGE, HMGB1, S100B, S100A8 and S100A9 by RT–PCR (**A**), western blotting (**B, C**) and immunoistochemical staining (**D**) on the lungs of C57BL6 mice infected with *Aspergillus* conidia intranasally at different days postinfection (dpi). In **C**, S100B was assessed on BAL by western blotting. For immunohistochemistry, lung sections were incubated overnight with anti–S100B or anti–RAGE antibody followed by the secondary antibodies. Nuclei were counter–stained with DAPI. (**E**) Lung sections from transgenic mice expressing *s100b*–EGFP+ at different days postinfection. Bars, 100 µm. Microscopy was performed on a DM Rb epifluorescence microscope equipped with a digital camera. Representative of 2 experiments.

We assessed the expression of RAGE in the lungs of mice infected with *Aspergillus* conidia by immunohistochemical staining, protein and gene expression analysis. RAGE expression was observed at mRNA (**Fig. 1A** and **Fig. S1A**) and protein (**Fig. 1B**) levels and maximally occurred in alveolar epithelial cells, as revealed by immunofluorescence staining (**Fig. 1D**). On assessing which putative ligands of RAGE were concomitantly expressed in infection, we found that HMGB1 was not increased either at the level of gene (**Fig. 1A** and **Fig. S1A**) or protein (**Fig. 1B**) expression. In contrast, S100B was promptly induced in infection, and declined thereafter to return to basal levels a week later, as revealed by gene and protein expression analysis in the lung (**Fig. 1A,B** and **Fig. S1A**) and protein secretion in the bronchoalveolar lavage fluid (BAL) (**Fig. 1C**). S100B immunoreactivity was high in bronchiolar epithelial cells as revealed in wild–type mice (WT) (immunofluorescence staining in **Fig. 1D**) or in transgenic mice expressing *s100b*–EGFP+ (**Fig. 1E**). Further analysis on purified lung cells from transgenic mice confirmed that epithelial cells were major sources of S100B in infection (**Fig. S1B**) while *Ager* was expressed on epithelial cells, macrophages, dendritic cells (DCs) (**Text S1**) and polymorphonuclear neutrophils (PMNs) (**Fig. S1C**), as described [5]. Presumably associated with PMNs' infiltration, *s100a8* and *s100a9* expressions in the lung mainly occurred at 3 days post–infection (**Fig. 1A**). These data suggest that S100B pairs with RAGE very early in infection before its transcriptional downregulation. The prompt induction of S100B followed by its downregulation suggests that S100B may serve as a danger signal to control inflammation.

### RAGE–deficient mice develop pathogen–induced inflammation

**Figure 2:**
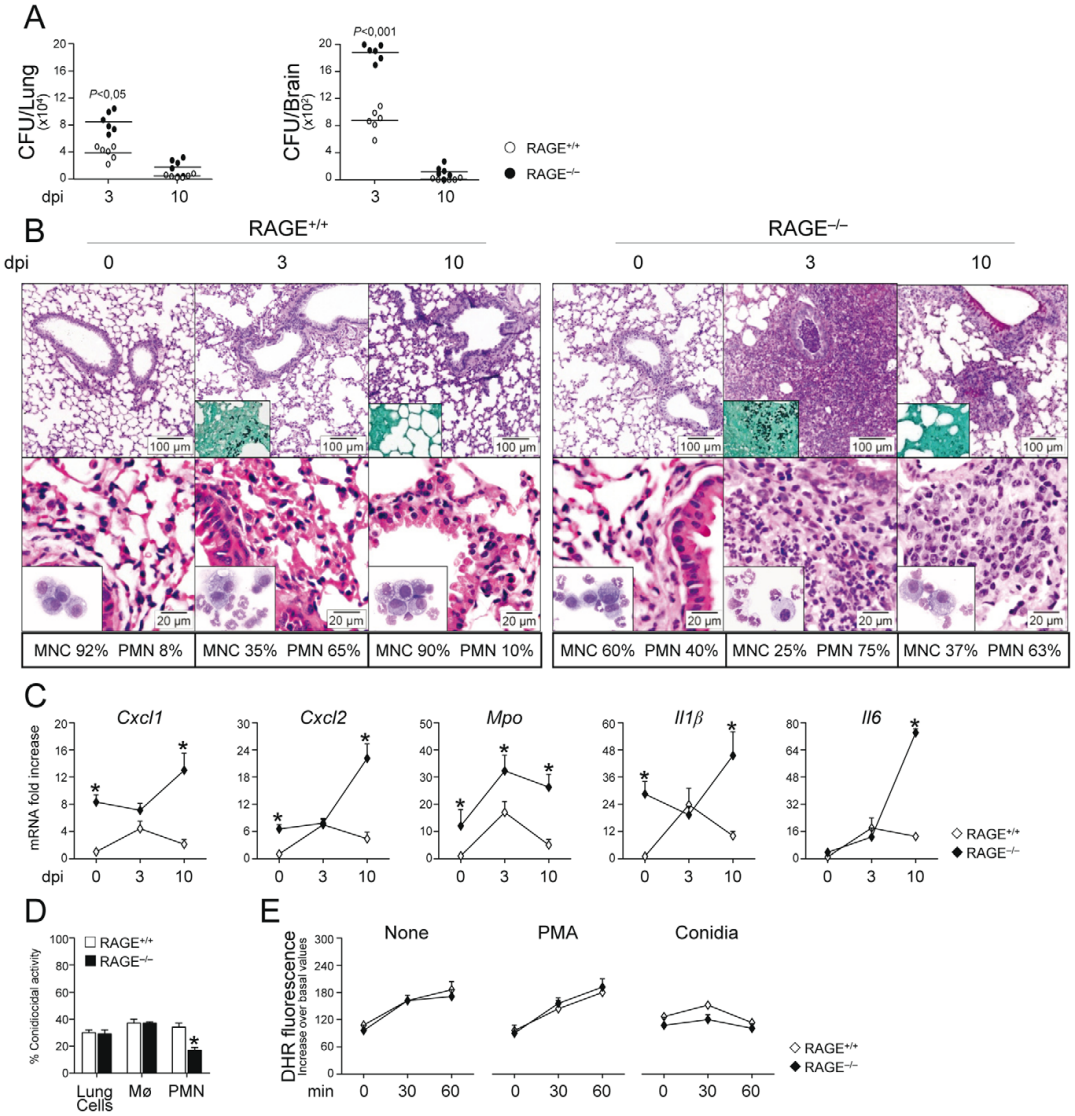
RAGE–deficient mice develop pathogen–induced inflammation. Fungal growth (CFU±SE) (**A**), lung histology (PAS, H&E and Gomori stainings) and BAL morphometry (**B**), inflammatory chemokines and cytokine gene expression in the lungs by real–time RT–PCR (**C**), conidiocidal activity (**D**), oxidant production (**E**) in mice infected with live *Aspergillus* conidia intranasally. Note the sustained parenchymal damage (PAS staining in the upper panel of **Fig. 2B**), fungal growth (Gomori staining in the inset), and inflammatory cell recruitment in lungs (H&E staining in the lower panel) and BAL (May–Grünwald Giemsa–staining in the inset) in RAGE KO mice. Bars indicated magnifications. Dpi, days postinfection. BAL morphometry (numbers refer to % polymorphonuclear (PMN) or mononuclear (MNC) cells), lung RT–PCR, conidiocidal activity and oxidant production were assessed 3 days after the infection. Total lung cells, purified alveolar macrophages and PMNs were incubated with unopsonized resting conidia at 37°C for conidiocidal activity [percentage of colony forming units' inhibition (mean ± SE) at 60 min] or oxidant production by DHR. PMA, phorbol 12–myristate 13–acetate. *P, KO vs WT mice. Data are pooled from 4 experiments or representative of 2 experiments (for histology).

To determine the role of the S100B/RAGE axis in response to the fungus, we evaluated parameters of infection, inflammation and adaptive immunity in RAGE KO mice with pulmonary aspergillosis. Despite an initial higher fungal growth in the lung and brain of KO than WT mice, the fungal growth was eventually restrained in both types of mice (**Fig. 2A**). Inflammation and signs of parenchyma damage, in contrast, were greatly exacerbated in RAGE KO mice and failed to resolve as opposed to WT mice (**Fig. 2B**, upper panels with fungi magnified in the inset). The number of PMNs increased and maintained elevated in the lung parenchyma and the BAL fluids (**Fig. 2B**, lower panels and inset) of RAGE KO mice. Gene expression analysis of the lung confirmed the higher and persistent inflammatory response in KO than WT mice, as revealed by the higher mRNA expression of *Cxcl1*, *Cxcl2* and *Mpo* genes as well as genes for inflammatory cytokines, such as IL–1β and IL–6 (**Fig. 2C**).

Despite the fact that an inflammatory response was not observed upon challenge with inactivated conidia (data not shown), the failure to resolve inflammation was not secondary to either a deficient conidiocidal activity of lung cells, including macrophages (**Fig. 2D**), or a defective oxidant production (**Fig. 2E**). PMNs only from KO mice showed between 25 to 35% reduction of their conidiocidal activity as compared to WT mice, a finding pointing to a requirement for RAGE in the execution of PMNs' effector activity. These data indicate that RAGE, known to mediate PMN recruitment through interaction with beta 2 integrins [26], is neither required for lung inflammatory cell recruitment or oxidant production in aspergillosis but unexpectedly protects from unintended inflammation.

Both the subverted innate inflammatory response to the fungus [25] and the requirement for RAGE in DC and T cell functions [27], [28] would predict altered adaptive Th responses to the fungus. This was indeed the case as shown by the results of lung DC and Th cell activation in response to the fungus. Purified DCs from RAGE KO mice responded to *Aspergillus* conidia or hyphae with higher expression level of mRNA for IL–1β, IL–6, IL–23 (p19), and similar levels of IL–12 (p35) or IL–10 compared to WT DCs (**Fig. S2A**). Of interest, similar to the response to the fungus, higher levels of inflammatory cytokines were also observed in KO vs WT DCs in response to the TLR2/TLR6 ligand bacterial lipopeptide macrophage–activating lipopeptide (MALP–2) but not to LPS or ODN–CpG (**Fig. S2B**). In terms of Th cell activation, although cytokine and transcription factor mRNAs were higher in unstimulated CD4+T cells from KO than WT mice, a further increased was observed for Th2 (*Gata3/Il4*) or Th17 b(*Rorc/Il17a*) but not for Th1 (*Tbet/Ifnγ*) or Treg (*Foxp3/Il10*) specific transcripts (**Fig. S2C**). Thus, RAGE deficiency is associated with deregulated innate and adaptive antifungal immunity and the inflammatory program activated in DCs in response to the fungus/TLR ligands is compatible with the impairment of antifungal Th1/Treg protective responses and upregulation of inflammatory Th2/Th17 cell responses [29], [30].

### RAGE pairs with S100B for anti–inflammatory and pro–inflammatory signals

**Figure 3:**
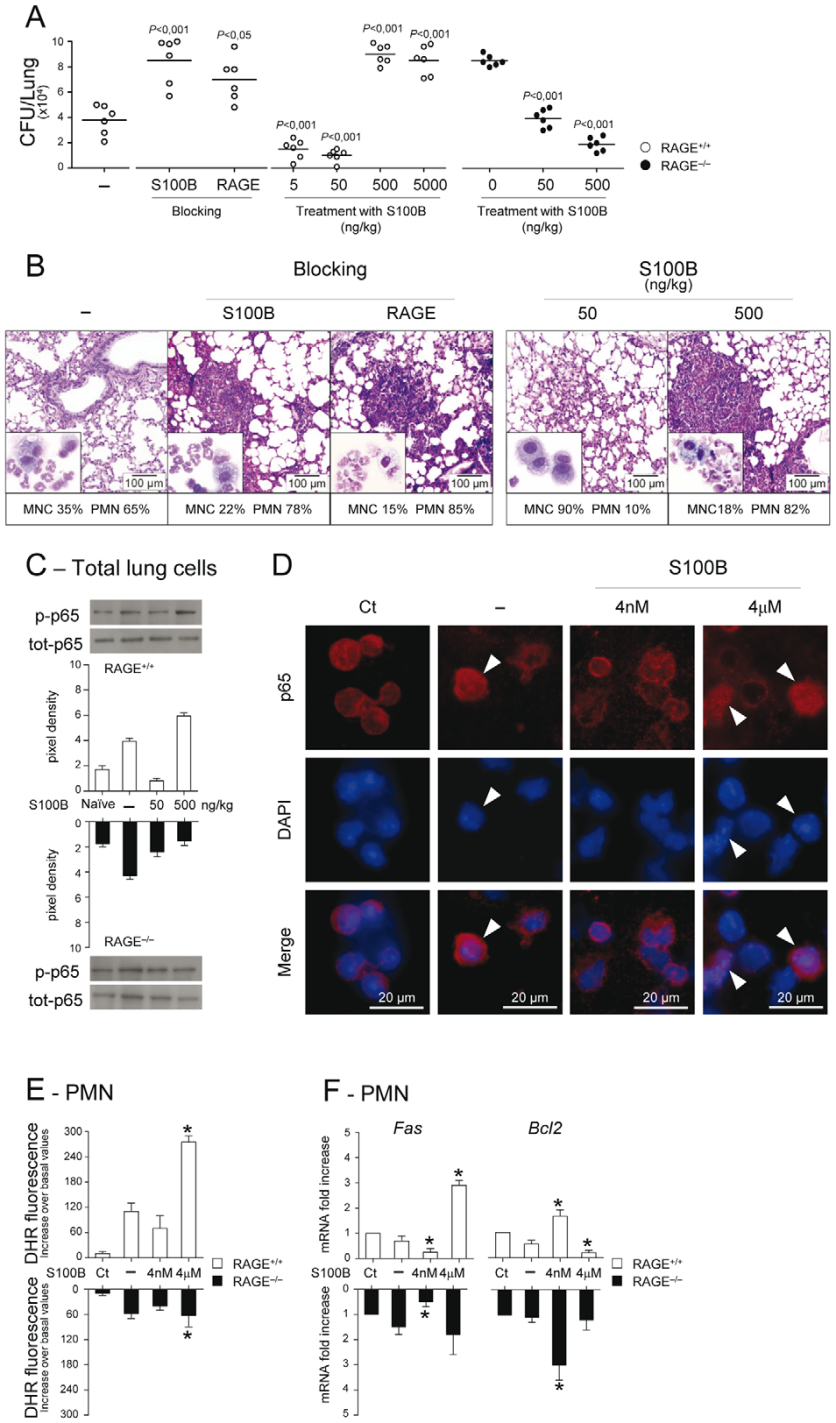
Effects of S100B administration or neutralization on aspergillosis. Fungal growth (CFU±SE) (**A**), lung histology (PAS staining) and BAL morphometry (May–Grünwald Giemsa–staining in the inset) (**B**) in C57BL6 or RAGE KO mice infected with *Aspergillus* live conidia intranasally and treated intraperitoneally for 3 consecutive days with different doses (ng/kg) S100B, 1 mg/kg anti–S100B or 0.5 mg/kg anti–RAGE antibodies. *P*, treated *vs* untreated (–) mice. NF–κB activation was assessed by western blotting (**C**) on lung cells from WT and KO mice, untreated (–) or treated with 50 or 500 ng/kg S100B, a day after the last treatment, or by nuclear translocation (indicated by arrowheads in **D**) on purified PMNs, unexposed (Ct, control) or exposed in vitro for 60 min to *Aspergillus* conidia alone (–) or in the presence of S100B at 4 nM or 4 µM. Western blotting data are presented as immunoblots of cell lysates with phosphorylation–specific antibodies and fold increases (pixel density) in the phosphorylated to total protein ratios. (**E**) Oxidant production was assessed PMNs exposed as in D (by DHR at 60 min). (**F**) *Fas* and *Bcl2* expressions were assessed by real–time RT–PCR after 6 h–exposure. Representative of 3 experiments. *P<0.05, treated *vs* untreated (–) cells.

To formally prove that RAGE pairs with S100B in infection, experiments of S100B neutralization or administration were performed. We found that S100B neutralization decreased resistance to infection in WT mice as indicated by the increased fungal growth (**Fig. 3A**), PMN recruitment and inflammation in the lung (**Fig. 3B**), an effect that was mimicked by treatment with antibodies neutralizing RAGE engagement (**Fig. 3A,B**). Accordingly, exogenously administered S100B decreased the fungal growth and the inflammatory pathology, but this occurred at nanomolar doses ranging from 5 to 50 ng/kg but not at doses up to 5000 ng/kg (**Fig. 3A,B**). Both effects were RAGE–dependent being abrogated, albeit partially for low–dose S100B, in RAGE KO mice (**Fig. 3A**). Similar experiments done for HMGB1 (**Text S1**) showed that the fungal burden and inflammation were both increased upon its administration and involved RAGE (**Fig. S3**). Because S100B itself didn't show a direct activity on fungal growth and morphology (**Text S1** and **Fig. S4**) and was ineffective if given before the infection (data not shown), these data suggest that S100B pairs with RAGE for anti– and pro–inflammatory activities, a feature consistent with the unique ability of S100B to exhibit opposite effects depending on doses [12], [17], [31].

Mechanistically, we assessed whether S100B affected the activation of nuclear factor κB (NF–κB) and oxidant production, important inflammatory pathways downstream RAGE activation [3], [4] in vivo and in vitro on purified PMNs, known to respond to S100B [12]. In vivo, 500, but not 50, ng/kg S100B promoted RAGE–dependent NF–κB activation in the lung (**Fig. 3C**). In vitro, micromolar but not nanomolar S100B activated NF–κB (**Fig. 3D**) and increased oxidant production in response to the fungus (**Fig. 3E**). Interestingly, and consistent with the dose–dependent prosurvival/prodeath effects of S100B on cells [32], *Fas* expression was decreased and antiapoptotic *Bcl2* expression increased in WT and KO PMNs exposed to nanomolar S100B and the opposite was true with micromolar S100B acting via RAGE (**Fig. 3F**). These data confirm that RAGE activation by S100B is dependent on doses and also suggest that S100B may possess characteristics beyond the RAGE activating function which mediate its anti–inflammatory effects.

### The S100B/RAGE axis restrains TLR2/MyD88–dependent inflammation

**Figure 4:**
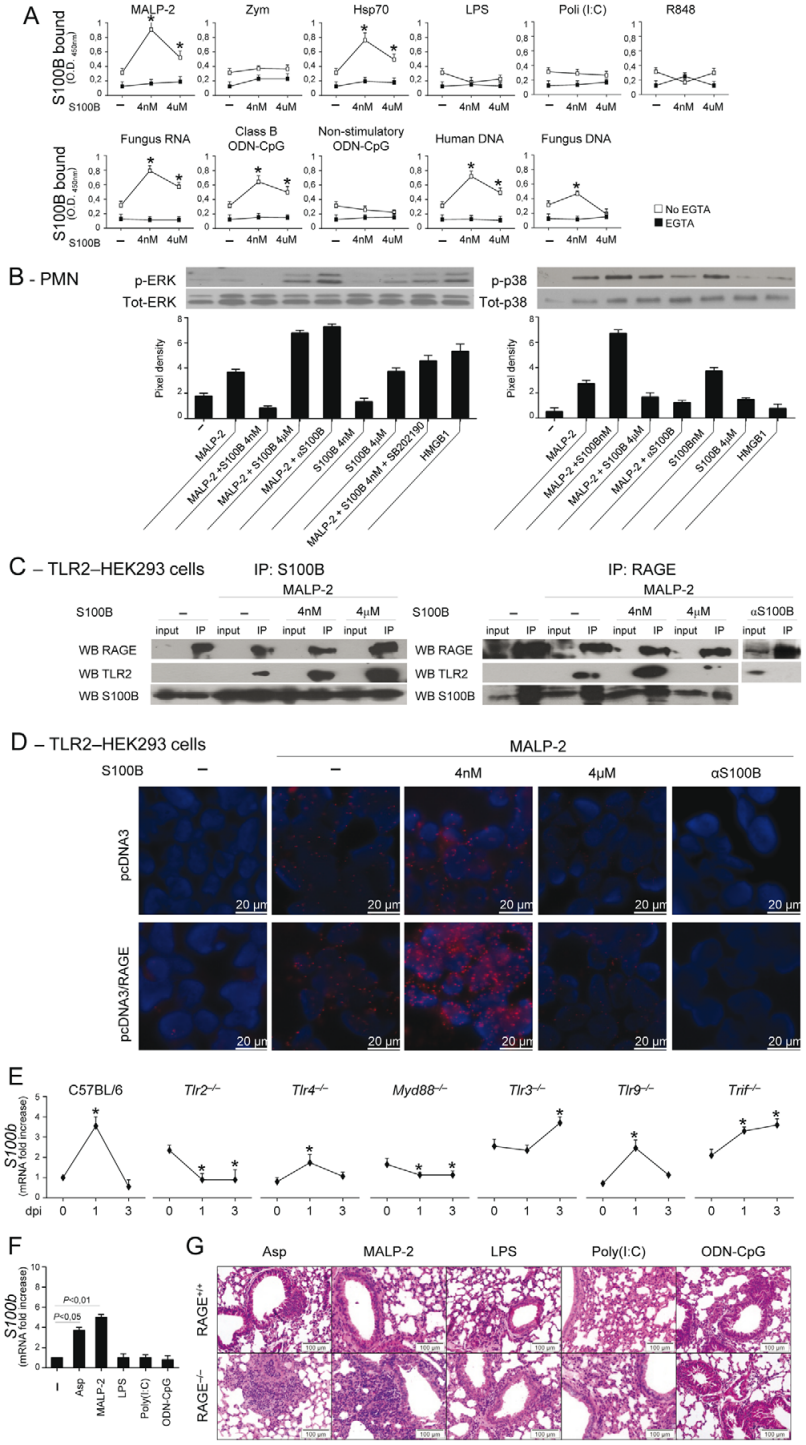
The S100B/RAGE axis restrains TLR2/MyD88–dependent inflammation. (**A**) Binding of S100B to TLR ligands by ELISA. S100B was incubated in microtiter plates coated with 10 µg/ml (as by preliminary experiments) of MALP–2, Zymosan, HSP70, LPS, Poly(I:C), fungal DNA or RNA, mammalian genomic DNA, Class B ODN–CpG (2006), nonstimulatory ODN–CpG (2310) and R–848 with and without 1 mM EGTA. Data indicate the mean ±SE of triplicates from three independent experiments. *P<0.05, S100B vs no S100B (–). (**B**) Levels of ERK1/2 or p38 MAPK (30 min) in purified PMNs exposed to 5 µg/ml MALP–2, nanomolar or micromolar S100B, 20 µg/ml anti–S100B antibody, 5 µM SB202190, 300 nM HMGB1, alone or in combination, for 30 min as described. Data are presented as immunoblots of cell lysates with phosphorylation–specific antibodies and fold increases (pixel density) in the phosphorylated to total protein ratios. Representative of 2 experiments. (**C**) TLR2-transfected HEK293 cells were stimulated with MALP–2 for 30 min with and without 4 nM or µM S100B or 20 µg/ml anti–S100B antibody. Cell lysates were subjected to immunoprecipitation after overnight incubation with 2 µg/ml polyclonal anti–S100B or anti–RAGE antibody. Immunoprecipitates were probed with antibodies to the corresponding antigens. RT-PCR analysis confirmed that both RAGE and S100B were expressed on tranfected HEK293 cells and experiments in KO cells confirmed the specificity of the anti-RAGE antibody (data not shown). Representative of 2 experiments. (**D**) Direct visualization of RAGE interaction with TLR2 in the presence of nanomolar S100B by in situ proximity ligation assay (PLA). TLR2-transfected HEK293 cells were transiently transfected with a RAGE expression vector (pcDNA3/RAGE) or empty vector (pcDNA3) and stimulated with MALP–2 for 30 min with or without 4 nM or µM S100B or 20 µg/ml anti–S100B antibody. Cells were stained with anti-RAGE and anti-TLR2 antibodies, and subjected to PLA. (**E**) *s100b* gene–expression by real–time RT–PCR in different TLR-deficient mice at different days postinfection (dpi) with *Aspergillus* conidia intranasally. *P<0.05, dpi 1 and 3 *vs* 0. (**F**) *S100b* gene–expression by real–time RT–PCR in lung of C57BL6 mice injected with 2.5 µg MALP–2, 10 µg LPS, 50 µg Poly(I:C), 50 µg Class B ODN–CpG 3 days before. *P*, treated *vs* untreated (–) mice. (**G**) H&E–stained sections from C57BL6 or RAGE KO mice injected as in **F**, 3 days before. Representative of 2 experiments.

Danger–sensing mechanisms are known to participate in the TLR responses to PAMPs [11], [20] and to negatively regulate excessive inflammation during infection [10]. Given the ability of HMGB1 to bind and act in synergy with endogenous and exogenous TLR ligands [11], [33], we assessed whether S100B also binds TLR ligands in solid phase by ELISA. We found that S100B highly binds exogenous and endogenous TLR ligands, such as MALP–2, HSP70, class B ODN–CpG (ODN 1982), mammalian DNA, fungal RNA and, partly, DNA, in a Ca^2+^–and dose–dependent manner, with the maximum binding activity observed at the nanomolar dose (**Fig. 4A**). No binding was observed to Zymosan, LPS, double–stranded RNA [polyinosinic–polycytidylic acid, Poly(I:C)], non-CpG ODN (ODN 1982) or single–stranded RNA (the imidazoquinoline resiquimod R848). These data suggest that S100B may interact with TLR2 (HSP70) but not with Dectin–1 (Zymosan), with the heterodimer TLR2/TLR6 (MALP–2) and with intracellular nucleic acid–sensing TLRs.

Because TLR2 activates the inflammatory state of PMNs in infection [34], [35] and unrestrained inflammation occurred in condition of defective S100B/RAGE axis, we hypothesized that the S100B/RAGE axis may inhibit TLR2/MyD88–driven inflammation to the fungus. We assessed therefore whether and how nanomolar or micromolar S100B would affect TLR2–mediated activation of PMNs. We found that ERK phosphorylation in response to MALP–2 was inhibited by nanomolar S100B and potentiated by micromolar S100B or by blocking serum S100B. This occurs through a p38–dependent mechanism, as shown by the ability of nanomolar S100B to induce p38–phosphorylation as well as ERK phosphorylation in the presence of the specific p38 inhibitor SB202190. Like micromolar S100B, HMGB1 activated ERK more than p38 phosphorylation (**Fig. 4B**). These data indicate that S100B, like HMGB1 [11], potentiates the biological activity of TLR2 ligands upon forming complexes with them.

However, they also unexpectedly revealed that forming complexes with nanomolar S100B negatively regulates their functions. That p38 is a negative regulator of TLR2 expression has already been described [36]. We assessed here whether inhibition of TLR2 occurred through physical association by performing immunoprecipitation studies with TLR2–transfected HEK cells stimulated with MALP–2, in the presence or not of S100B. Both RAGE and TLR2 were found to associate with nanomolar or micromolar S100B upon TLR2 stimulation, but TLR2 physically associated with RAGE only in the presence of nanomolar S100B (**Fig. 4C**). Although RAGE was found to be expressed in TLR2–transfected HEK 293 cells (unpublished observations), we transiently transfected TLR2–HEK 293 cells with RAGE to visualize the RAGE/TLR2 interaction using the in situ proximity ligation assay. We confirmed that RAGE strongly interacts with TLR2 in the presence of nanomolar but not micromolar S100B. In addition, the lack of interaction observed upon S100B neutralization suggests that endogenous S100B likely mediates TLR2/RAGE physical interaction in steady-state conditions (**Fig. 4D**). Thus, S100B interacts with RAGE and TLR2 and mediates the physical association of the two at nanomolar doses. Because S100B production mainly occurred in infection via the TLR2/MyD88 pathway (**Fig. 4E, F**), our findings indicate the existence of an autocrine/paracrine loop by which TLR2–induced S100B binds to extracellular RAGE to inhibit TLR2 upon physical association. This scenario would suggest an increased responsiveness to TLR2–mediated inflammation of RAGE KO mice. Consistent with the high reactivity of DCs to MALP–2 (**Fig. S2B**), the inflammatory response to intranasally delivered MALP–2 was higher in RAGE KO than WT mice as compared to other TLR agonists the sensitivity to which was not different between KO and WT mice (**Fig. 4G**).

### TLR3/9 signaling inhibits S100B expression via TRIF/noncanonical NF–κB

**Figure 5:**
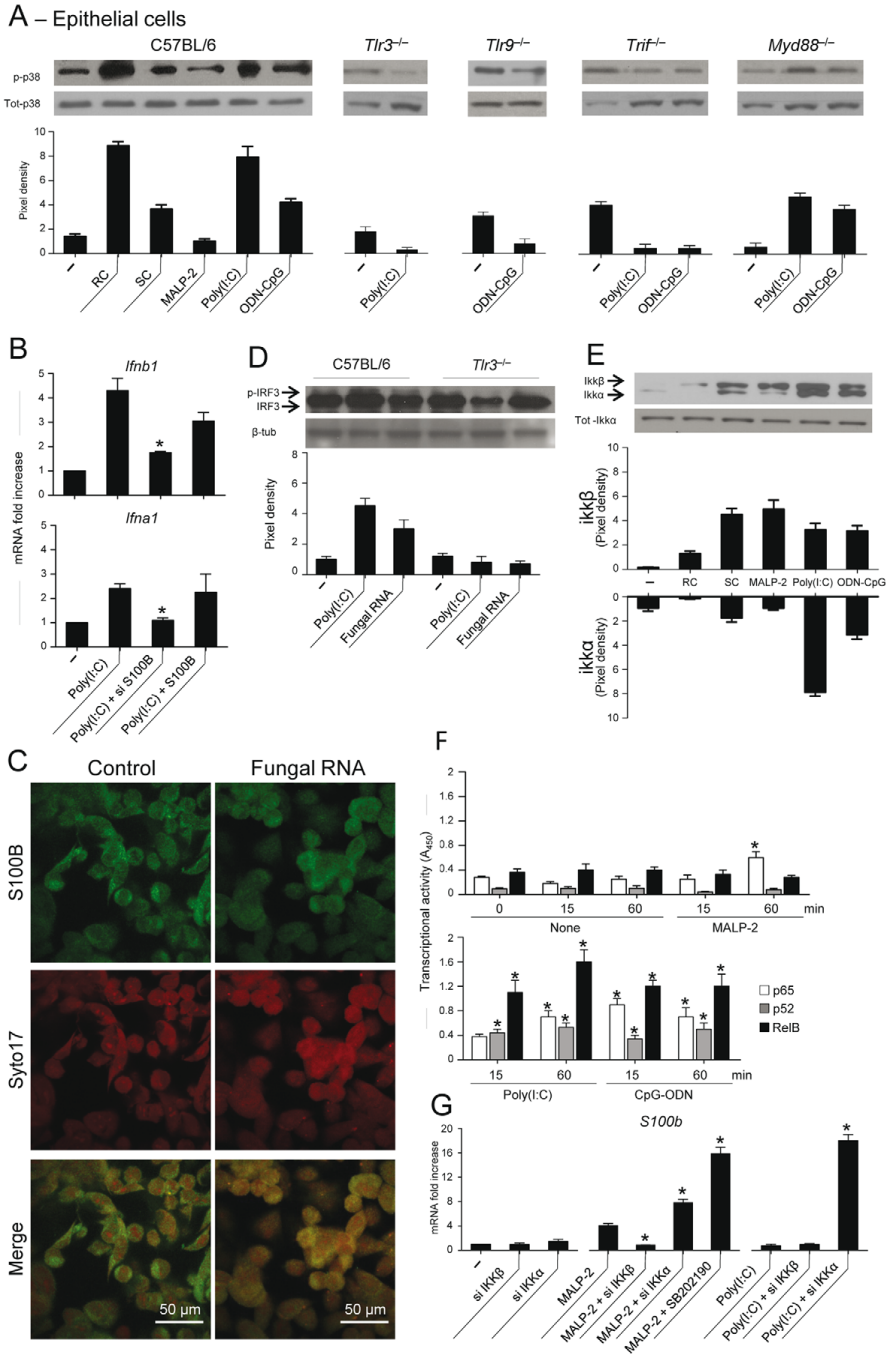
TLR3 and TLR9 inhibit *s100b* expression via TRIF/noncanonical NF–κB. (**A**) Levels of p38 phosphorylation on purified epithelial cells from C57BL6 mice or TLR KO mice, unexposed (–) or exposed to *Aspergillus* resting (RC) or swollen (SC) conidia, 5 µg/ml MALP–2, 10 µg/ml Poly(I:C) or 10 µg/ml ODN–CpG for 8 h. (**B**) *Ifnb1* or *Ifna1* gene expression by real time RT–PCR on epithelial cells from C57BL6 mice exposed to Poly(I:C) in the presence of siS100B or 4 nM S100B. *P<0.05, siRNA –treated *vs* untreated cells. Representative of 2 experiments. (**C**) S100B co-localization with fungal RNA in HEK293 cells pulsed with fungal RNA and stained with Syto17 red fluorescent nucleic acid stain and anti-S100B antibody followed by FITC-conjugated goat anti–rabbit. Mock-pulsed (control) and pulsed cells were analyzed on confocal microscope. (**D**) Fungal RNA stimulates epithelial cells via TLR3. Levels of IRF3 phosphorylation on lung epithelial cells exposed to Poly(I:C) or fungal RNA. Data are presented as immunoblots of cell lysates and fold increases (pixel density) in the phosphorylated to total protein ratios. (**E**) Immunoblots and pixel density of IKKβ and IKKα phosphorylation in epithelial cells from C57BL6 mice exposed as in (**A**). (**F**) Results from an ELISA procedure to monitor activation of p65, p52, and RelB in nuclear extract from epithelial cells exposed as in (**A**) for different lengths of time. Time 0 indicates untreated cells. Relative activities (A_450_) are mean±SE of two experiments, each in triplicate. *P<0.05, treated vs untreated cells. (**G**) *s100b* gene expression by real time RT–PCR upon canonical/noncanonical NF–κB inhibition by siRNA in epithelial cells exposed to MALP–2 or Poly(I:C), as above. SB202190 (5 µM) was used as p38 inhibitor. *P<0.05, siRNA–treated or p38–inhibited cells *vs* MALP–2 or Poly(I:C)–exposed cells. Representative of 2 experiments.

The finding that S100B production in vivo was upregulated in the absence of TLR3 and TRIF, conditions in which we noticed a defective transcriptional downregulation of S100B (**Fig. 4E**), led us to suppose that binding to intracellular nucleic acids is a mechanism by which S100B is down-regulated and its pro–inflammatory activity restrained in infection. We resorted to lung epithelial cells as major sources of S100B in infection. We assessed p38 phosphorylation in cells from WT and selected TLR–KO mice exposed to *Aspergillus* resting (RC) or swollen (SC) conidia, MALP–2, Poly(I:C) or ODN–CpG and the relative contribution of endogenous S100B. We found that p38 phosphorylation occurred maximally in response to *Aspergillus* RC and Poly(I:C), to a lesser extent in response to ODN–CpG and SC, did not occur in response to MALP–2, and was largely TLR3/TLR9/TRIF–dependent, but MyD88–independent (**Fig. 5A**). Both *Ifnb1* and *Ifna1* gene expression in response to Poly(I:C) were unaffected upon the addition of S100B but decreased upon neutralizing S100B by siRNA (**Fig. 5B**), a finding indicating that S100B participates in the functional sensing of intracellular nucleic acids by TLR3. Although similar results were obtained in response to ODN–CpG, the overall responsiveness of epithelial cells to TLR9 was lower (data not shown), as already reported [37]. In terms of source of intracellular nucleic acids, consistent with the binding activity of S100B in vitro, we found that fungal RNA not only complexes with S100B in infection (**Fig. 5C**) but also activates epithelial cells in a TLR3–dependent manner, as indicated by IRF3 phosphorylation (**Fig. 5D**).

The transcriptional downregulation of *s100b* in infection led us to hypothesize that transcription factors downstream p38/TRIF would mediate this effect. Given the existence of specific binding sites for NF–κB family members in the promoter of both human (GenBank: M59486) and murine (GenBank: NC_000076.5) *S100b*, we assessed whether NF–κB transcription factors regulate *s100b* gene expression. For this purpose, we evaluated the activation of canonical/noncanonical NF–κB pathways downstream TLR2/MyD88 and TLR3/TLR9/TRIF and their contribution to *s100b* gene expression. Of the two IkB kinase complex catalytic subunits, known to have opposing roles in inflammation [38], IKKβ more than IKKα was phosphorylated in response to SC, MALP–2, the opposite was true in response to Poly(I:C), while both pathways were activated by ODN–CpG (**Fig. 5E**). We also quantified the activation of the NF–κB family members, using an ELISA kit specific for mouse p65, p52 and RelB. Significant nuclear translocation occurred for p52 and RelB, but not for p65, following 15–60 min of exposure to Poly(I:C). Significant translocation of all members was observed in response to ODN–CpG, while only p65 translocation occurred in response to MALP–2 (**Fig. 5F**). The two pathways had opposite effects on *S100b* gene expression, as shown by experiments in which either pathway was silenced by siRNA. *S100b* expression was inhibited upon blocking the canonical pathway or promoted upon blocking the noncanonical, p38-dependent, pathway (**Fig. 5G**). These data suggest that *s100b* expression is transcriptionally regulated by the sequential action of downstream MyD88– and TRIF–dependent NF–κB signalling pathways.

### S100B activity in vivo is contingent upon TLRs

**Figure 6:**
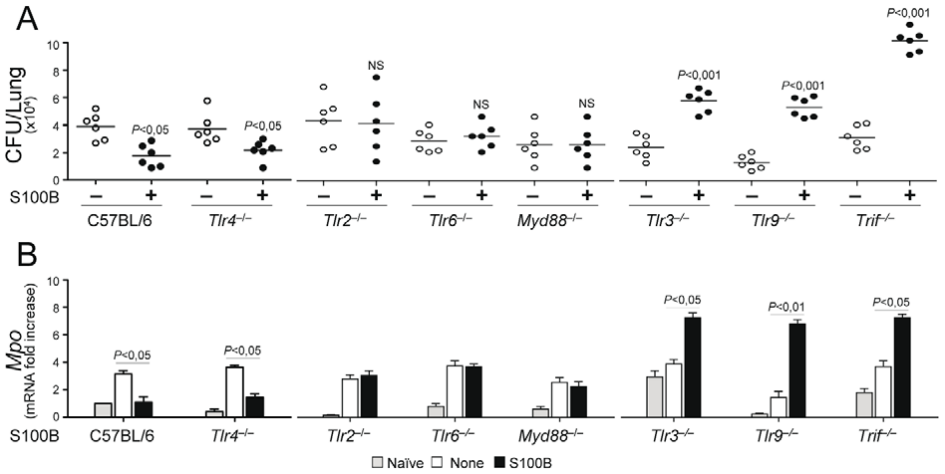
S100B activity *in vivo* is contingent upon TLR signaling. Fungal growth (CFU±SE) (**A**) and (**B**) *Mpo* expression by real time RT–PCR in the lung of TLR–deficient mice infected with *Aspergillus* conidia and treated with 50 ng/ml S100B as in legend to **Fig. 3**. Data were obtained at 3 days postinfection. Representative of 4 experiments. *P*, treated vs untreated (None) mice.

**Figure 7:**
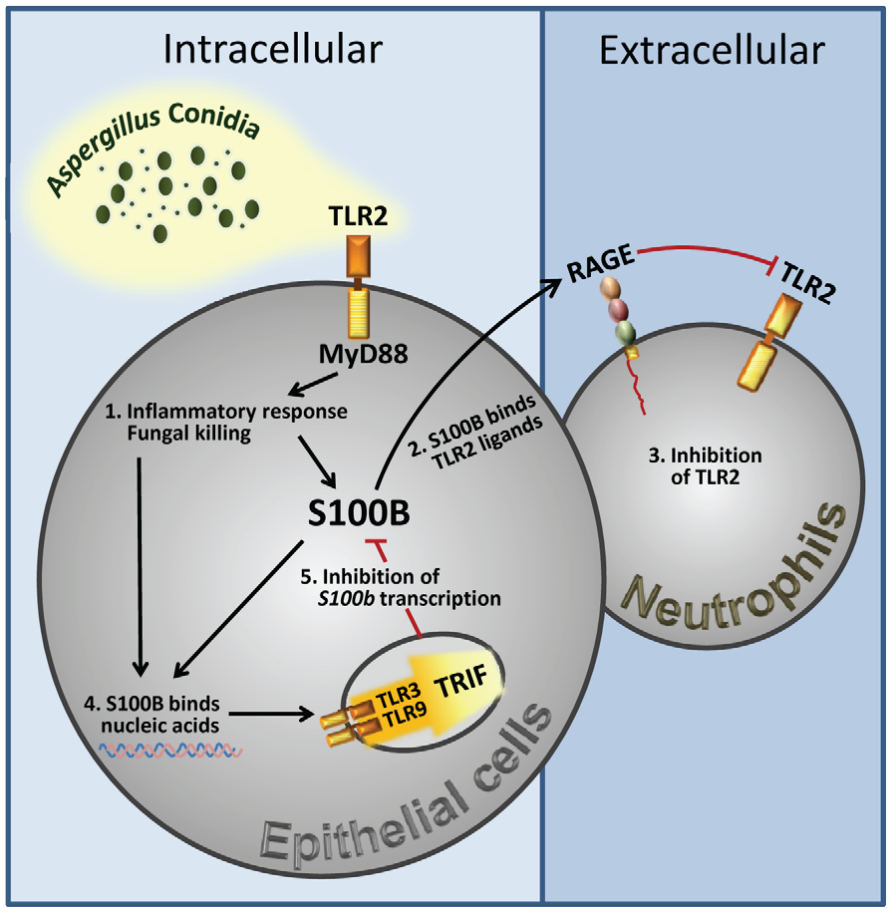
Spatiotemporal integration of signals from TLRs and RAGE by S100B limits pathogen– and danger–induced inflammation. The figure shows that TLR2 activation on epithelial cells by the fungus resulted in the release of the Ca^2+^–binding protein, S100B, that paracrinally binds to RAGE, on polymorphonuclear neutrophils, and mediates its association with TLR2 with subsequent inhibition. However, S100B, upon binding to nucleic acids, also activates an intracellular TLR3/TLR9/TRIF–dependent pathway culminating in the transcriptional down-regulation of S100B. The transcriptional regulation of S100B by the sequential action of downstream MyD88– and TRIF–dependent NF–κB signalling pathways provides the molecular basis for an evolving braking circuit in infection whereby the endogenous danger protects the host against pathogen–induced inflammation and a nucleic acid–sensing mechanism resolves danger–induced chronic inflammation.

Experiments in vivo confirmed that the pro- and anti-inflammatory activity of S100B is contingent upon these TLRs. The anti–inflammatory activity of nanomolar S100B, as revealed by the fungal growth restriction and PMN recruitment, occurred independently of TLR4 but required the presence of TLR2, TLR6 and the MyD88 adaptor. Consistent with the ability of the TLR3/TLR9/TRIF pathway to downregulate *s100b*, S100B became pro–inflammatory at the nanomolar dose in the relative absence of TLR9, TLR3 and the adaptor TRIF (**Fig. 6A, B**). These in vivo findings confirm that the spatiotemporal integration of signals from TLRs and RAGE by S100B limits pathogen– and danger–induced inflammation in murine aspergillosis (**Fig. 7**).

## Discussion

To initiate an appropriate inflammatory response, organisms have developed ways to recognize potentially life–threatening signals. Our study reveals that sequential signaling between different innate immune biosensors serves to limit pathogen– and danger– induced collateral inflammation in infection. This occurs through a previously undescribed TLR/RAGE interaction via S100B, an EF–hand calcium–binding protein, with both intra– and extracellular activities, that acts in either an autocrine or paracrine manner through RAGE [12], [15].

RAGE is known to interact with TLR9 via HMGB1 which results in either a potentiation [19] or suppression [39] of TLR9 function. We found that, upon engagement, RAGE associated with and inhibited TLR2. This occurred through epithelial cell–released S100B that paracrinally inhibited the TLR2–dependent activity of recruited PMNs, a finding consistent with the ability of RAGE to impair neutrophil functions [40] as well as with the down–regulated TLR2 activity in pulmonary aspergillosis [41]. PMNs' recruitment is a characteristic feature of pulmonary aspergillosis [23] and PMNs' activity is tightly regulated by TLRs [34]. RAGE was dispensable for PMNs' recruitment but potently regulated TLR2–induced MAPK kinase activation, NF–κB phosphorylation and survival, via low, but not high, doses of S100B. Notably, the ability of S100B to bind TLR2 also predicts an activity on TLR2 in a RAGE-autonomous fashion. Therefore, consistent with the biology of RAGE and its ligands [3], [42], their up–regulation exerted a proximal role in the inflammatory cascade. However, at least for extracellular S100B, the interaction with RAGE also served to limit pathogen–induced inflammation. Thus, S100B plays a dual role in infection, restraining the inflammatory response in the early response to pathogen, through a paracrine epithelial cells/PMNs braking circuit, but also contributing, similar to HMGB1, to excessive inflammation through feed–forward RAGE activation [26] and likely through additional TLR interactions.

The opposite effects on cells observed with low and high doses of S100B could be mechanistically explained by considering that calcium binding triggers structural changes in the S100 protein that allow interaction with target proteins as an octamer or a higher-order multimer form [16]. Trophic *vs* toxic effects are observed on neuronal cells in which nanomolar S100B stimulate neurite growth and promote survival, while micromolar levels result in inflammatory effects [32], [43]. Structural and biochemical data have provided evidence that octameric S100B is highly stable and triggers RAGE activation by receptor dimerisation resulting in high–affinity binding [15], [16] to the RAGE V and C(1) domains activating NF–κB [15]. Both domains are important for ligand binding and for intracellular signaling, respectively. In contrast, nanomolar S100B required RAGE to inhibit TLR2 for the elaboration of its anti–inflammatory activity. We have already shown that some S100B–induced cellular effects may not depend on RAGE signalling yet requiring the receptor [44]. This appears to be the case in our model in which the ability of S100B to bind endogenous and exogenous TLR2 ligands may offer a plausible molecular explanation for RAGE/TLR2 physical association. How this may prevent TLR2 signalling is not obviously clear, although signalling by TLR2 upon binding of ligands possessing fatty acyl moieties suggests a dynamic model of interaction, in which only a specific orientation of the ligand favors formation of a signal inducing ternary complex [45]. Thus, similar to HMGB1 [11], S100B, by forming complexes with various TLR ligands, may present the partner molecule to its normal receptor in a way in which the conformation of the partner molecule is changed or in an allosteric interaction with the receptor or both.

The ability of S100B to bind nucleic acids, while qualifying S100B as possible sentinel for nucleic acid–mediated immune activation [20], also serves to explain the intracellular function of S100B in epithelial cells in infection. It is known that, upon calcium binding, the change of conformation in the C–terminal domain of S100B allows the exposure of hydrophobic residues critical for the binding to a variety of target proteins [46], thereby affecting their activities and allowing the elaboration of a variety of intracellular functions [12]. We found that S100B was able to bind, in a calcium–dependent manner, Class B ODN–CpG, mammalian DNA and fungal RNA and DNA, resulting in the activation of a p38/TRIF–dependent signalling, downstream TLR3 and TLR9. Thus, intracellular S100B may signal trough both TLR3 and TLR9 to scavenge pathogen– and host–derived nucleic acids. Of interest, at variance with HMGB1 [19], S100B also discloses a TLR9–depending signalling pathway that converges on TRIF rather than on MyD88 [7]. The molecular basis for this result in epithelial cells is presently under investigation, but is consistent with the finding that modification of the structure of the DNA ligand affects its sub–cellular localization and this may impact on sorting and signaling adapters as well as the biological response to TLR9 activation in DCs [47], [48]. That S100B may affect the intracellular compartmentalization of DNA upon binding pathogen and self DNA is, ultimately, a likely expectation for a chaperon molecule that localizes to the cytoplasm in a soluble form and in complexes with cytoskeletal and filament–associated target proteins [31]. This may also predict an inherent risk of autoimmunity associated with S100B. Incidentally, elevated levels of S100B have been observed in certain immuno–mediated diseases [12].

In addition to binding fungus DNA, whose unmethylated CpG motifs activates TLR9 [49], S100B also bound fungal RNA, a PAMP able to activate DCs for antifungal priming [50]. That endogenous mRNA [51] and pathogen RNA [52] activate TLR3 is an established finding. We found that endogenous S100B binds fungal RNA and activation of epithelial cells by fungal RNA is TLR3–dependent. Thus, in addition to sensing tissue necrosis [53], TLR3, abundantly expressed on epithelial cells [37], functions as an endogenous sensor of fungal RNA. Even more interesting is the finding that the activation of the TRIF–dependent, nucleic acid sensing pathway, mainly considered an inducer of antimicrobial innate immune responses, contributes to resolution of inflammation in infection. This occurs by downregulating *s100b* gene expression transcriptionally via noncanonical NF–κB signalling. Although *s100b* gene expression is tightly regulated in human cells [54], little is known about mechanisms regulating its transcription. The transcriptional regulation of *s100b* expression by the sequential action of downstream MyD88– and TRIF–dependent NF–κB signalling pathways is thus a novel finding that not only establishes a link between pathogen– and danger–sensing signaling pathways but also confirms the inhibitory role of TLR3 on the S100B/RAGE axis [55].

*In toto*, we have identified a mechanism that discriminates between pathogen– and danger–induced immune responses via the spatiotemporal integration of signals from different innate immune biosensors. Conceptually, our study details an evolving braking circuit in infection whereby an endogenous danger protects the host against pathogen–induced inflammation and a nucleic acid–sensing mechanism terminates danger–induced inflammation. Thus, in addition to the notion that danger signal may terminate overactive immune responses [10], our study reveals that a pathogen–induced signal may also terminate unnecessary danger–induced injury. This raises the intriguing possibility that the host may have developed mechanisms to ameliorate the response to DAMPs via PAMPs. The scenario is dominated by the highly adaptive S100B/RAGE axis that, in sensing danger, plays a critical and unanticipated role as a fine modulator of inflammation via the promiscuous activity of S100B at the extracellular and intracellular levels. On a translational level, our findings suggest that a defective danger sensing associated with the different isoforms of the RAGE receptor may underlie individual differences in the clinical course of invasive aspergillosis and the inherent patient's susceptibility to infection.

## Materials and Methods

### Ethics statement

Experiments were performed according to the Italian Approved Animal Welfare Assurance A–3143–01. Legislative decree 157/2008-B regarding the animal licence obtained by the Italian Ministry of Health lasting for three years (2008–2011). Infections were performed under avertin anesthesia and all efforts were made to minimize suffering.

### Mice

Female C57BL6 mice, 8–10 wk old, mice were purchased from Charles River (Calco, Italy). Homozygous *Tlr2–/–, Tlr3–/–, Tlr4–/–, Tlr9–/–, Myd88–/– and Trif–/–* mice on a C57BL6 background were bred under specific pathogen–free conditions at the Animal Facility of Perugia University, Perugia, Italy. RAGE*–/–* mice were obtained from Dr. Angelika Bierhaus (Heidelberg, Germany). *s100b*–EGFP+ transgenic mice [56] were obtained from Dr. Catherine Legraverend (Montpellier, France).

### Fungal strains, infections, and treatments

The strain of *A. fumigatus* was obtained as described [25]. Viable resting, swollen *Aspergillus* conidia and hyphae were obtained as described [30]. For infection, mice were anesthetized by intraperitoneal (i.p.) injection of 2.5% avertin (Sigma Chemical Co, St. Louis, MO) before instillation of a suspension of 2×10^7^ conidia/20 µl saline intranasally (i.n.). Fungi were suspended in endotoxin–free (Detoxi–gel; Pierce, Rockford, IL) solutions (<1.0 EU/mL, as determined by the LAL method). Mice were monitored for fungal growth [Colony forming units (CFU 9/organ, mean ± SE]. BAL was performed by cannulating the trachea and washing the airways with 3 ml of PBS to collect the BAL fluid. Total and differential cell counts were done by staining BAL smears with May–Grünwald Giemsa reagents (Sigma) before analysis. At least 200 cells per cytospin preparation were counted and the absolute number of each cell type was calculated. Photographs were taken using a high–resolution Microscopy Olympus DP71 (Olympus, Milan, Italy). Mice were treated daily i.p. for 3 consecutive days starting the day of the infection with different doses of purified S100B (see below), 1 mg/kg polyclonal rabbit anti–S100B antibodies (Swant, CH–6501 Bellinzona, Switzerland) or 0.5 mg/kg anti–RAGE goat polyclonal IgG (Santa Cruz Biotechnology, inc. DBA, Milan, Italy). Control received PBS or isotype controls (Sigma–Aldrich).

### In vivo treatments with TLR agonists

MALP–2 (2.5 µg), Poly(I:C) (50 µg), ultrapure LPS from *Salmonella minnesota* Re 595 (10 µg) (all from Sigma Chemical Co) and Class B ODN–CpG (50 µg) [24] were given once intranasally to mice infected as above. Control received PBS or isotype controls (Sigma–Aldrich). Mice were sacrificed three days after treatment for histology (H&E staining) and *s100b* expression by real–time RT–PCR. Control received PBS.

### Histology, fluorescence and immunohistochemistry

For histology, sections of paraffin–embedded tissues were stained with the periodic acid–Schiff (PAS), hematoxylin and eosin (H&E) or Gomori's methenamine Silver procedures [25]. For detecting S100B–expressing cells, lungs in OCT or purified cells from *s100b*–EGFP mice were analyzed. For immunohistochemistry, lung sections were incubated overnight with polyclonal anti–S100B antibody (1∶100) or polyclonal anti–RAGE antibody (1∶20) followed by the secondary antibodies, i.e., tetramethyl rhodamine isocyanate–conjugated goat anti–rabbit IgG (Sigma–Aldrich) for S100B, and AlexaFluor 594 donkey anti–goat IgG (Invitrogen), for RAGE. Nuclei were counter–stained with 4′,6-diamidino-2-phenylindole (DAPI). Endogenous peroxidase activity was quenched using 3% H2O2 in PBS. Immunostaining of lungs from RAGE KO mice was used as negative controls. Fluorescence and immunofluorescence microscopy was performed on a DM Rb epifluorescence microscope equipped with a digital camera (Leica, Wetzlar, Germany).

### Cell preparation, cultures and treatments

Purified lung CD11b^+^Gr–1^+^ PMNs (>98% pure on FACS analysis) were obtained as described [34]. Lung epithelial cells, at ∼99% expressing cytokeratin, on pan–cytokeratin antibody staining of cytocentrifuge preparations, and >90% viable on trypan blue exclusion assay, were isolated as described [57].The average yield of tracheal cells was 1.7×10^5^ cells/trachea [±0.58×10^5^ (SD)]. Alveolar macrophages were purified by plastic adherence. Total lung cells, purified alveolar macrophages and PMNs were incubated with unopsonized resting conidia at 1∶1 ratio at 37°C for conidiocidal activity [percentage of colony forming units inhibition (mean ± SE) at 60 min] or oxidant production [oxidation of dihydrorhodamine 123 (DHR), Molecular Probes (Invitrogen S.R.L. San Giuliano Milanese, Milan, Italy, measured by fluorimetry with the multifunctional microplate reader Tecan Infinite 200, Tecan Austria GmbH, Salzurg, Austria) at different time points. PMNs or epithelial cells were exposed to nanomolar or micromolar S100B as described [58], 20 µg/ml anti–S100B antibody (SWant), 300 nM HMGB1, 5 µg/ml MALP–2, 10 µg/ml Poly(I:C), 10 µg/ml ultrapure LPS from *Salmonella minnesota* Re 595 and 10 µg/ml ODN–CpG. In vitro experiments were done in the presence of 2% FBS. Control cells were treated with PBS, DMSO or control antibody.

### S100B binding assays

S100B binding to TLR ligands was assessed in solid phase by ELISA. Briefly, plates were coated overnight at 4°C with 10 µg/ml (based on preliminary experiments) of MALP–2, Zymosan (Sigma Aldrich), HSP70 (StressMarq Biosciences Inc, Victoria Canada), Poly(I:C) or LPS in carbonate buffer (pH 9.55) or total fungal DNA or RNA, human DNA, Class B ODN–CpG (2006), non-CpG ODN (ODN 1982) [24], resiquimod (R–848, Invivogen, Labogen S.r.l. Rho, Italy) in Reacti–Bind^TM^ DNA Coating Solution (Pierce). Fungal DNA and RNA were obtained as described [50]. Total fungal RNA was routinely pretreated with RNase–free DNase I (50 units of DNase I/100 mg RNA) (Sigma Aldrich) at 25°C for 2 h. Nanomolar or micromolar S100B was added in blocking buffer (TBS 1%BSA) for 2 h at room temperature followed by the addition of rabbit anti–S100B antibodies (1∶1000) and HRP–conjugated rabbit secondary antibody (R&D Systems, Space Import–Export srl Milano, Italy). EGTA was used at 1 mM. The plates were developed using TMB Microwell Peroxidase Substrate system (BioFX Laboratories, Inc MD, U/SA). ODs were read at 450 nm. Data indicate the mean ±SE of triplicates from three independent experiments.

### Syto17 red fluorescent nucleic acid stain

To detect S100B co-localization with fungal RNA, TLR2–transfected HEK293 cells were pulsed with fungal RNA by means of N-[1-(2,3-dioleoyloxypropyl]-N,N,N,-trimethylammonium methylsulfate (DOTAP; Roche), as described [50]. After pulsing cells were fixed in 3.7% formaldehyde, Triton–X100 permeabilized and incubated with Syto17 red fluorescent universal nucleic acid stain (Molecular Probe; 2.5 µM, 5 min) and anti-S100B antibody (1∶20 dilution) followed by FITC-conjugated goat anti–rabbit IgG (Vector Laboratories). Mock-pulsed (control) and pulsed cells were analyzed on confocal microscope Nikon Eclipse TE-2000U (Tokyo, Japan).

### Western blotting

Blots of cells lysates were incubated with monoclonal rabbit monoclonal anti–S100B IgG (clone EP1576Y, Epitomics, CA), goat polyclonal anti–RAGE IgG (Santa Cruz Biotechnology, Inc.), rabbit anti–HMGB1 IgG2a (Calbiochem, Milan, Italy), mouse monoclonal anti–TLR2 IgG2a, Santa Cruz Biotechnology, Inc.), rabbit polyclonal Abs recognizing the unphosphorylated form of ERK and p38 followed by horseradish peroxidase–conjugated anti–goat, mouse or rabbit IgG (Cell Signaling Technology) or biotin–conjugated (Vectastain Elite ABC system; Vector Laboratories, Burlingame, CA, USA) secondary antibodies. Blots were developed with the Enhanced Chemiluminescence detection kit (Amersham Pharmacia Biotech, Milan, Italy) and SuperSignal West Pico (Pierce). Scanning densitometry was done on a Scion Image apparatus. The pixel density of bands was normalized against total proteins or tubulin. The inhibitor p38 (5 µM, SB202190) was purchased from Calbiochem (San Diego, CA) and dissolved at 1000× the final concentration in DMSO (Sigma). Control experiments included staining without the primary antibody.

### Co–immunoprecipitation

The human HEK293 embryonic kidney cell lines stably transfected with human TLR2 were maintained as described [35]. Cells were stimulated with MALP–2 for 30 min with and without 4 nM or µM S100B or 20 µg/ml anti–S100B antibodies (SWant). Cell lysates were subjected to immunoprecipitation after overnight incubation with 2 µg/ml polyclonal anti–S100B (SWant) or anti–RAGE (Santa Cruz Biotechnology, Inc) antibody. Immunoprecipitates were probed with antibodies to the corresponding antigens. Control experiments included western blottings on immunoprecipated with an irrelevant antibody.

### In situ proximity ligation assay (PLA)

We resorted to PLA [59] to directly visualize the RAGE interaction with TLR2 in an S100B–dependent manner. TLR2–transfected HEK293 cells were transiently transfected with a RAGE expression vector (pcDNA3/RAGE) or empty vector (pcDNA3) and stimulated with MALP–2 for 30 min with or without 4 nM or µM S100B or 20 µg/ml anti–S100B antibody (SWant). Cells were then fixed in cold methanol and treated with a rabbit anti–RAGE (H300, Santa Cruz Biotechnology, Inc) and a goat anti–TLR2 antibody, and subjected to PLA (OLINK Bioscience, Uppsala) according to the manufacturer's instructions. Cells were visualized on the DM Rb epifluorescence microscope.

### Canonical and noncanonical NF–κB

To detect NF–κB (p65) nuclear translocation, purified PMNs were fixed in cold methanol, permeabilized with Triton-X100 0.1% in PBS, incubated with blocking solution (PBS containing 3% BSA and 1% glycine), and incubated overnight at 4°C with rabbit anti-p65 (C-20) antibody (sc-372, Santa Cruz Biotechnology; 1∶50 dilution) followed by tetramethyl rhodamine isocyanate-conjugated goat anti-rabbit IgG (Sigma-Aldrich; 1∶50 dilution) as secondary antibody. Nuclei were counter-stained with DAPI. Cells were visualized on the epifluorescence microscope. We used an ELISAbased TransAM Flexi NFkB Family Kit (Active Motif) to monitor activity of NF–κB family members. Anti–phospho–IKKα (Ser180)/IKKβ (Ser181) rabbit Abs (Cell Signaling Technology) were used for western blotting of phospho IKKα and IKKβ. Western blotting with specific polyclonal antibodies (Santa Cruz Biotechnology) was done to assess level of p65.

### Exposure of epithelial cells to fungal RNA

Epithelial cells were exposed to fungal RNA (25 µg/ml) [50] for 8 h before determination of levels of IRF3 phosphorylation by immunoblotting with rabbit polyclonal anti–IRF3 antibodies and anti–rabbit–horseradish peroxidase (Santa Cruz Biotechnology Inc.). Data are presented as immunoblots of cell lysates and fold increases (pixel density) in the phosphorylated to total protein ratios.

### Expression and purification of S100B

Recombinant bovine S100B, 97% identical to mouse S100B, was expressed and purified as reported [31], [32]. Purified S100B was passed through END–X B15 Endotoxin Affinity Resin column to remove contaminating bacterial endotoxin. The S100B concentration was calculated using the M_r_ of the S100B dimer (21 kDa).

### SiRNA synthesis and transfection

SiRNA to target IKKα, IKKβ and S100B were done as described [30]. The siRNA specific sequences were selected, synthesized and annealed by the manufacturer, and were used in combination with nontargeted control siRNA (Ambion, Applied Biosystem International, Monza Italy). Transfections of siRNA (at 1 nM/well) were performed by using the INTERFERin^TM^Transfection reagent, as per manufacturer's instructions (PEQLAB Biotechnologie GmbH, Erlangen, Germany). Cells were stimulated 48 h after transfection at 37°C. Expression of IKKα, IKKβ and S100B transcripts in transfected cells was evaluated by RT–PCR or western blotting.

### Reverse transcriptase–PCR and real–time PCR

Real–time RT–PCR was performed using the iCycler iQ detection system (Bio–Rad) and SYBR Green chemistry (Finnzymes Oy, Espoo, Finland). Cells were lysed and total RNA was extracted using RNeasy Mini Kit (QIAGEN, Milan, Italy) and was reverse transcribed with Sensiscript Reverse Transcriptase (QIAGEN) according to the manufacturer's directions. The sense/antisense primers were as follows: *Ager* sense 5′–GCCCTCATTGATGTCTTCCACC–3′; antisense (5′–GAACTCATGGCAGGCCGTGGTC–3′); *s100b* sense 5′–GCCCTCATTGATGTCTTCCACC–3′; antisense 5′–GAACTCATGGCAGGCCGTGGTC–3′; *s100a8* sense 5′–TCGTGACAATGCCGTCTGAACTG–3′; antisense 5′–TGCTACTCCTTGTGGCTGTCTTTG–3′; *s100a9*, sense 5′– CGCAGCATAACCACCATCATC–3′; antisense 5′–GCCATCAGCATCATACACTCC–3′; *Hmgb1* sense, 5′–GGCTGACAAGGCTCGTTATG–3′; antisense 5′–GCAACATCACCAATGGATAAGC–3′;*Fas* sense 5′–CTACTGCGATTCTCCTGGCTGTG–3′; antisense 5′–AGTTTGTATTGCTGGTTGCCTGTGC–3′; *Bcl2* sense 5′–ACGAGTGGGATGCTGGAGATG–3′; antisense 5′–TCAGGCTGGAAGGAGAGATGC–3′. Other primers were as described [30] Amplification efficiencies were validated and normalized against *Gapdh*. The thermal profile for SYBR Green real time PCR was at 95°C for 3 min, followed by 40 cycles of denaturation for 30 s at 95°C and an annealing/extension step of 30 sec at 60°C. Each data point was examined for integrity by analysis of the amplification plot. The mRNA–normalized data were expressed as relative cytokine mRNA in stimulated cells compared to that of mock–infected cells.

### Statistical analysis

Data were analyzed by GraphPad Prism 4.03 program (GraphPad Software, San Diego, CA). Student's *t* test or analysis of variance (ANOVA) and Bonferroni's test were used to determine the statistical significance (*P*) of differences in organ clearance and in vitro assays. The data reported are either from one representative experiment out of three to five independent experiments (western blotting and RT–PCR) or pooled from three to five experiments, otherwise. The in vivo groups consisted of 6–8 mice/group.

## Supporting Information

Figure S1 RAGE and DAMPs expression in pulmonary aspergillosis. (A) Expression of Ager, hmgb1, s100b, s100a8 and s100a9 by real time RT-PCR on lung of C57BL6 mice at different days postinfection (dpi) with Aspergillus conidia intranasally. Representative of 2 experiments. (B) S100B-expression on purified cells from transgenic mice expressing s100b-EGFP+ infected with Aspergillus conidia 3 days before. M∅ alveolar macrophages, DC, dendritic cells, PMN, polymorphonuclear neutrophils. (C) Expression of Ager by RT-PCR on purified lung cells from uninfected C57BL6 mice. Microscopy was performed on a DM Rb epifluorescence microscope equipped with a digital camera. Representative of 2 experiments.
(0.41 MB TIF)

Figure S2 RAGE-deficient mice develop pathogen-induced Th inflammation. (A) Purified DCs from uninfected mice were exposed to live resting conidia or hyphae as described for 18 h before real time RT-PCR. (B) Cytokine gene-expression by real-time RT-PCR in DCs from RAGE KO or WT uninfected mice exposed to MALP-2, LPS or ODN-CpG for 18 h. (C) Freshly isolated CD4+T cells from TLN were assessed for transcription factor expression by RT-PCR. P, KO vs WT mice. P, KO vs WT mice. Data are pooled from 4 experiments or representative of 2 experiments (for histology). Representative of 2 experiments. P, KO vs WT DCs. N.D., not determined.
(0.57 MB TIF)

Figure S3 Effects of HMGB1 administration in mice with aspergillosis. Fungal growth (CFU±SE) (A) and lung histology (PAS staining) (B) in C57BL6 or RAGE KO mice infected with Aspergillus live conidia intranasally and treated intraperitoneally for 3 consecutive days with 50 µg/kg HMGB1. P, treated vs untreated (-) mice. Representative of 3 experiments. *P<0.05, treated vs untreated (-) cells.
(0.65 MB TIF)

Figure S4 Effect of S100B on A. fumigatus morphology and germination. Germination refers to the percentages (mean ± SE) of germinating cells over a total of 400 cells counted. Magnification x 40. Shown are the pooled results from 2 experiments. Photographs were taken using a high Resolution Microscopy Color Camera AxioCam, using the AxioVision Software Rel. 3.1 (Carl Zeiss S.p.A., Milano, Italy).
(1.25 MB TIF)

Text S1 Supplemental methods referred to Supplemental Figures S1, S3, S4.
(0.04 MB DOC)

## References

[1] Gallucci S, Matzinger P (2001). Danger signals: SOS to the immune system.. Curr Opin Immunol.

[2] Janeway CA, Medzhitov R (2002). Innate immune recognition.. Annu Rev Immunol.

[3] Donato R (2007). RAGE: a single receptor for several ligands and different cellular responses: the case of certain S100 proteins.. Curr Mol Med.

[4] Schmidt AM, Yan SD, Yan SF, Stern DM (2001). The multiligand receptor RAGE as a progression factor amplifying immune and inflammatory responses.. J Clin Invest.

[5] Sparvero LJ, Asafu-Adjei D, Kang R, Tang D, Amin N (2009). RAGE (Receptor for Advanced Glycation Endproducts), RAGE ligands, and their role in cancer and inflammation.. J Transl Med.

[6] Lin L (2006). RAGE on the Toll Road?. Cell Mol Immunol.

[7] O'Neill LA (2006). How Toll-like receptors signal: what we know and what we don't know.. Curr Opin Immunol.

[8] Chen GY, Tang J, Zheng P, Liu Y (2009). CD24 and Siglec-10 selectively repress tissue damage-induced immune responses.. Science.

[9] Liu Y, Chen GY, Zheng P (2009). CD24-Siglec G/10 discriminates danger- from pathogen-associated molecular patterns.. Trends Immunol.

[10] Sitkovsky MV, Ohta A (2005). The ‘danger’ sensors that STOP the immune response: the A2 adenosine receptors?. Trends Immunol.

[11] Bianchi ME (2009). HMGB1 loves company.. J Leukoc Biol.

[12] Donato R, Sorci G, Riuzzi F, Arcuri C, Bianchi R (2009). S100B's double life: intracellular regulator and extracellular signal.. Biochim Biophys Acta.

[13] Gebhardt C, Riehl A, Durchdewald M, Nemeth J, Furstenberger G (2008). RAGE signaling sustains inflammation and promotes tumor development.. J Exp Med.

[14] Kalea AZ, Reiniger N, Yang H, Arriero M, Schmidt AM (2009). Alternative splicing of the murine receptor for advanced glycation end-products (RAGE) gene.. Faseb J.

[15] Leclerc E, Fritz G, Weibel M, Heizmann CW, Galichet A (2007). S100B and S100A6 differentially modulate cell survival by interacting with distinct RAGE (receptor for advanced glycation end products) immunoglobulin domains.. J Biol Chem.

[16] Ostendorp T, Leclerc E, Galichet A, Koch M, Demling N (2007). Structural and functional insights into RAGE activation by multimeric S100B.. Embo J.

[17] Leclerc E, Fritz G, Vetter SW, Heizmann CW (2009). Binding of S100 proteins to RAGE: an update.. Biochim Biophys Acta.

[18] Ivanov S, Dragoi AM, Wang X, Dallacosta C, Louten J (2007). A novel role for HMGB1 in TLR9-mediated inflammatory responses to CpG-DNA.. Blood.

[19] Tian J, Avalos AM, Mao SY, Chen B, Senthil K (2007). Toll-like receptor 9-dependent activation by DNA-containing immune complexes is mediated by HMGB1 and RAGE.. Nat Immunol.

[20] Yanai H, Ban T, Wang Z, Choi MK, Kawamura T (2009). HMGB proteins function as universal sentinels for nucleic-acid-mediated innate immune responses.. Nature.

[21] Clynes R, Moser B, Yan SF, Ramasamy R, Herold K (2007). Receptor for AGE (RAGE): weaving tangled webs within the inflammatory response.. Curr Mol Med.

[22] van Zoelen MA, Schouten M, de Vos AF, Florquin S, Meijers JC (2009). The receptor for advanced glycation end products impairs host defense in pneumococcal pneumonia.. J Immunol.

[23] Segal BH (2009). Aspergillosis.. N Engl J Med.

[24] Aimanianda V, Bayry J, Bozza S, Kniemeyer O, Perruccio K (2009). Surface hydrophobin prevents immune recognition of airborne fungal spores.. Nature.

[25] Romani L, Fallarino F, De Luca A, Montagnoli C, D'Angelo C (2008). Defective tryptophan catabolism underlies inflammation in mouse chronic granulomatous disease.. Nature.

[26] Chavakis T, Bierhaus A, Al-Fakhri N, Schneider D, Witte S (2003). The pattern recognition receptor (RAGE) is a counterreceptor for leukocyte integrins: a novel pathway for inflammatory cell recruitment.. J Exp Med.

[27] Manfredi AA, Capobianco A, Esposito A, De Cobelli F, Canu T (2008). Maturing dendritic cells depend on RAGE for in vivo homing to lymph nodes.. J Immunol.

[28] Moser B, Desai DD, Downie MP, Chen Y, Yan SF (2007). Receptor for advanced glycation end products expression on T cells contributes to antigen-specific cellular expansion in vivo.. J Immunol.

[29] Zelante T, De Luca A, Bonifazi P, Montagnoli C, Bozza S (2007). IL-23 and the Th17 pathway promote inflammation and impair antifungal immune resistance.. Eur J Immunol.

[30] Bonifazi P, D'Angelo C, Zagarella S, Zelante T, Bozza S (2010). Intranasally delivered siRNA targeting PI3K/Akt/mTOR inflammatory pathways protects from aspergillosis.. Mucosal Immunol.

[31] Donato R (2003). Intracellular and extracellular roles of S100 proteins.. Microsc Res Tech.

[32] Huttunen HJ, Kuja-Panula J, Sorci G, Agneletti AL, Donato R (2000). Coregulation of neurite outgrowth and cell survival by amphoterin and S100 proteins through receptor for advanced glycation end products (RAGE) activation.. J Biol Chem.

[33] Hreggvidsdottir HS, Ostberg T, Wahamaa H, Schierbeck H, Aveberger AC (2009). The alarmin HMGB1 acts in synergy with endogenous and exogenous danger signals to promote inflammation.. J Leukoc Biol.

[34] Bellocchio S, Moretti S, Perruccio K, Fallarino F, Bozza S (2004). TLRs govern neutrophil activity in aspergillosis.. J Immunol.

[35] Moretti S, Bellocchio S, Bonifazi P, Bozza S, Zelante T (2008). The contribution of PARs to inflammation and immunity to fungi.. Mucosal Immunol.

[36] Sahay B, Patsey RL, Eggers CH, Salazar JC, Radolf JD (2009). CD14 signaling restrains chronic inflammation through induction of p38-MAPK/SOCS-dependent tolerance.. PLoS Pathog.

[37] Sha Q, Truong-Tran AQ, Plitt JR, Beck LA, Schleimer RP (2004). Activation of airway epithelial cells by toll-like receptor agonists.. Am J Respir Cell Mol Biol.

[38] Bonizzi G, Karin M (2004). The two NF-kappaB activation pathways and their role in innate and adaptive immunity.. Trends Immunol.

[39] Popovic PJ, DeMarco R, Lotze MT, Winikoff SE, Bartlett DL (2006). High mobility group B1 protein suppresses the human plasmacytoid dendritic cell response to TLR9 agonists.. J Immunol.

[40] Collison KS, Parhar RS, Saleh SS, Meyer BF, Kwaasi AA (2002). RAGE-mediated neutrophil dysfunction is evoked by advanced glycation end products (AGEs).. J Leukoc Biol.

[41] Chai LY, Kullberg BJ, Vonk AG, Warris A, Cambi A (2009). Modulation of Toll-like receptor 2 (TLR2) and TLR4 responses by *Aspergillus fumigatus*.. Infect Immun.

[42] Bierhaus A, Humpert PM, Morcos M, Wendt T, Chavakis T (2005). Understanding RAGE, the receptor for advanced glycation end products.. J Mol Med.

[43] Huttunen HJ, Fages C, Rauvala H (1999). Receptor for advanced glycation end products (RAGE)-mediated neurite outgrowth and activation of NF-kappaB require the cytoplasmic domain of the receptor but different downstream signaling pathways.. J Biol Chem.

[44] Adami C, Bianchi R, Pula G, Donato R (2004). S100B-stimulated NO production by BV-2 microglia is independent of RAGE transducing activity but dependent on RAGE extracellular domain.. Biochim Biophys Acta.

[45] Kajava AV, Vasselon T (2010). A network of hydrogen bonds on the surface of TLR2 controls ligand positioning and cell signaling.. J Biol Chem.

[46] Heizmann CW, Fritz G, Schafer BW (2002). S100 proteins: structure, functions and pathology.. Front Biosci.

[47] Guiducci C, Ott G, Chan JH, Damon E, Calacsan C (2006). Properties regulating the nature of the plasmacytoid dendritic cell response to Toll-like receptor 9 activation.. J Exp Med.

[48] Honda K, Ohba Y, Yanai H, Negishi H, Mizutani T (2005). Spatiotemporal regulation of MyD88-IRF-7 signalling for robust type-I interferon induction.. Nature.

[49] Ramirez-Ortiz ZG, Specht CA, Wang JP, Lee CK, Bartholomeu DC (2008). Toll-like receptor 9-dependent immune activation by unmethylated CpG motifs in *Aspergillus fumigatus* DNA.. Infect Immun.

[50] Bozza S, Perruccio K, Montagnoli C, Gaziano R, Bellocchio S (2003). A dendritic cell vaccine against invasive aspergillosis in allogeneic hematopoietic transplantation.. Blood.

[51] Kariko K, Ni H, Capodici J, Lamphier M, Weissman D (2004). mRNA is an endogenous ligand for Toll-like receptor 3.. J Biol Chem.

[52] Aksoy E, Zouain CS, Vanhoutte F, Fontaine J, Pavelka N (2005). Double-stranded RNAs from the helminth parasite Schistosoma activate TLR3 in dendritic cells.. J Biol Chem.

[53] Cavassani KA, Ishii M, Wen H, Schaller MA, Lincoln PM (2008). TLR3 is an endogenous sensor of tissue necrosis during acute inflammatory events.. J Exp Med.

[54] Castets F, Griffin WS, Marks A, Van Eldik LJ (1997). Transcriptional regulation of the human S100 beta gene.. Brain Res Mol Brain Res.

[55] Cai W, He JC, Zhu L, Lu C, Vlassara H (2006). Advanced glycation end product (AGE) receptor 1 suppresses cell oxidant stress and activation signaling via EGF receptor.. Proc Natl Acad Sci U S A.

[56] Vives V, Alonso G, Solal AC, Joubert D, Legraverend C (2003). Visualization of S100B-positive neurons and glia in the central nervous system of EGFP transgenic mice.. J Comp Neurol.

[57] You Y, Richer EJ, Huang T, Brody SL (2002). Growth and differentiation of mouse tracheal epithelial cells: selection of a proliferative population.. Am J Physiol Lung Cell Mol Physiol.

[58] Sorci G, Riuzzi F, Agneletti AL, Marchetti C, Donato R (2003). S100B inhibits myogenic differentiation and myotube formation in a RAGE-independent manner.. Mol Cell Biol.

[59] Soderberg O, Gullberg M, Jarvius M, Ridderstrale K, Leuchowius KJ (2006). Direct observation of individual endogenous protein complexes in situ by proximity ligation.. Nat Methods.

