# Supplementary figures and images for: The Danger Signal S100B Integrates Pathogen– and Danger–Sensing Pathways to Restrain Inflammation
Source: PLoS Pathog. 2011 Mar 10;7(3):e1001315. doi: 10.1371/journal.ppat.1001315 (PMC3053348; doi:10.1371/journal.ppat.1001315)

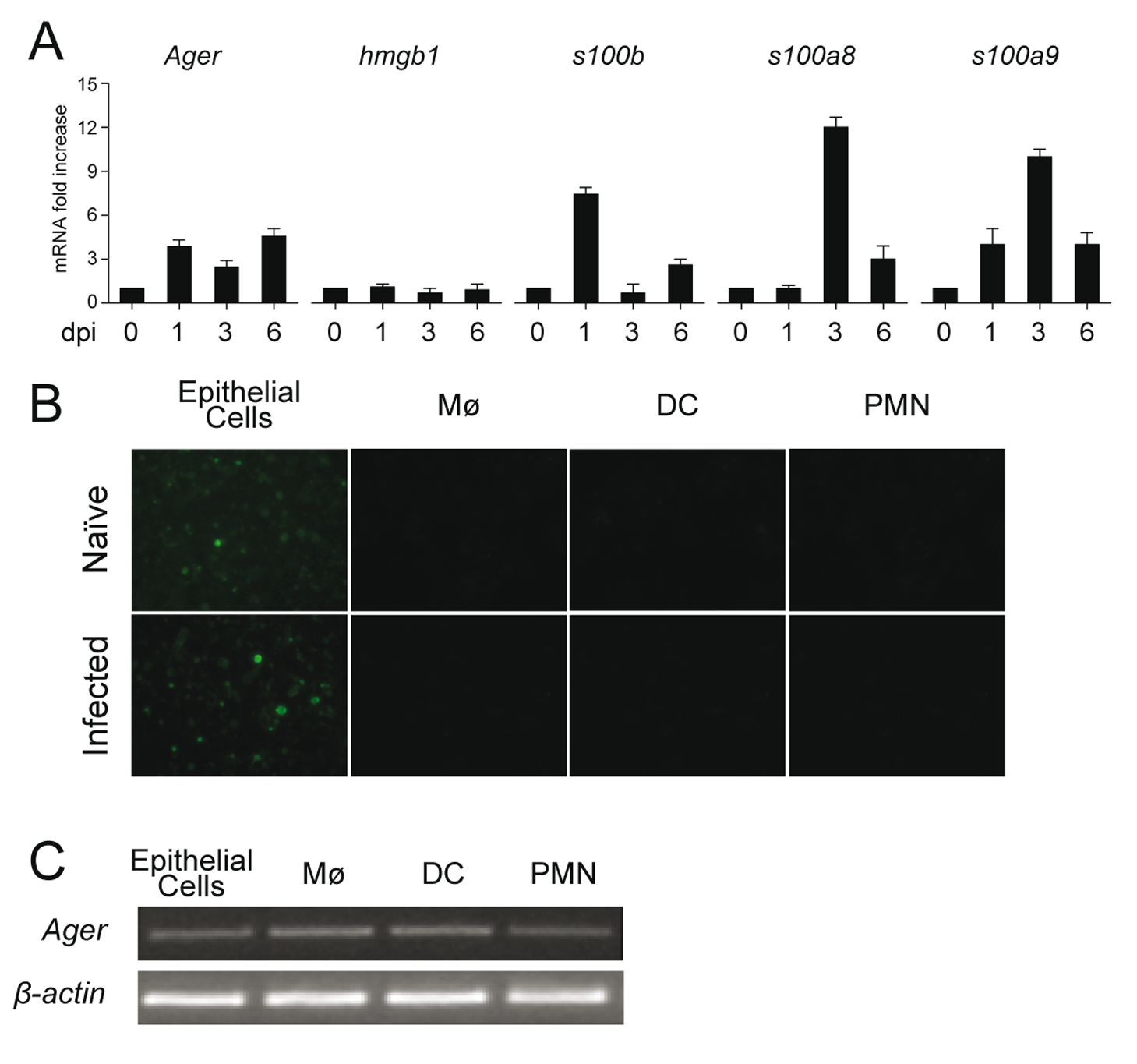

Supplement: Figure S1 — RAGE and DAMPs expression in pulmonary aspergillosis. (A) Expression of Ager, hmgb1, s100b, s100a8 and s100a9 by real time RT-PCR on lung of C57BL6 mice at different days postinfection (dpi) with Aspergillus conidia intranasally. Representative of 2 experiments. (B) S100B-expression on purified cells from transgenic mice expressing s100b-EGFP+ infected with Aspergillus conidia 3 days before. M∅ alveolar macrophages, DC, dendritic cells, PMN, polymorphonuclear neutrophils. (C) Expression of Ager by RT-PCR on purified lung cells from uninfected C57BL6 mice. Microscopy was performed on a DM Rb epifluorescence microscope equipped with a digital camera. Representative of 2 experiments. (0.41 MB TIF) [file ppat.1001315.s001.tif]

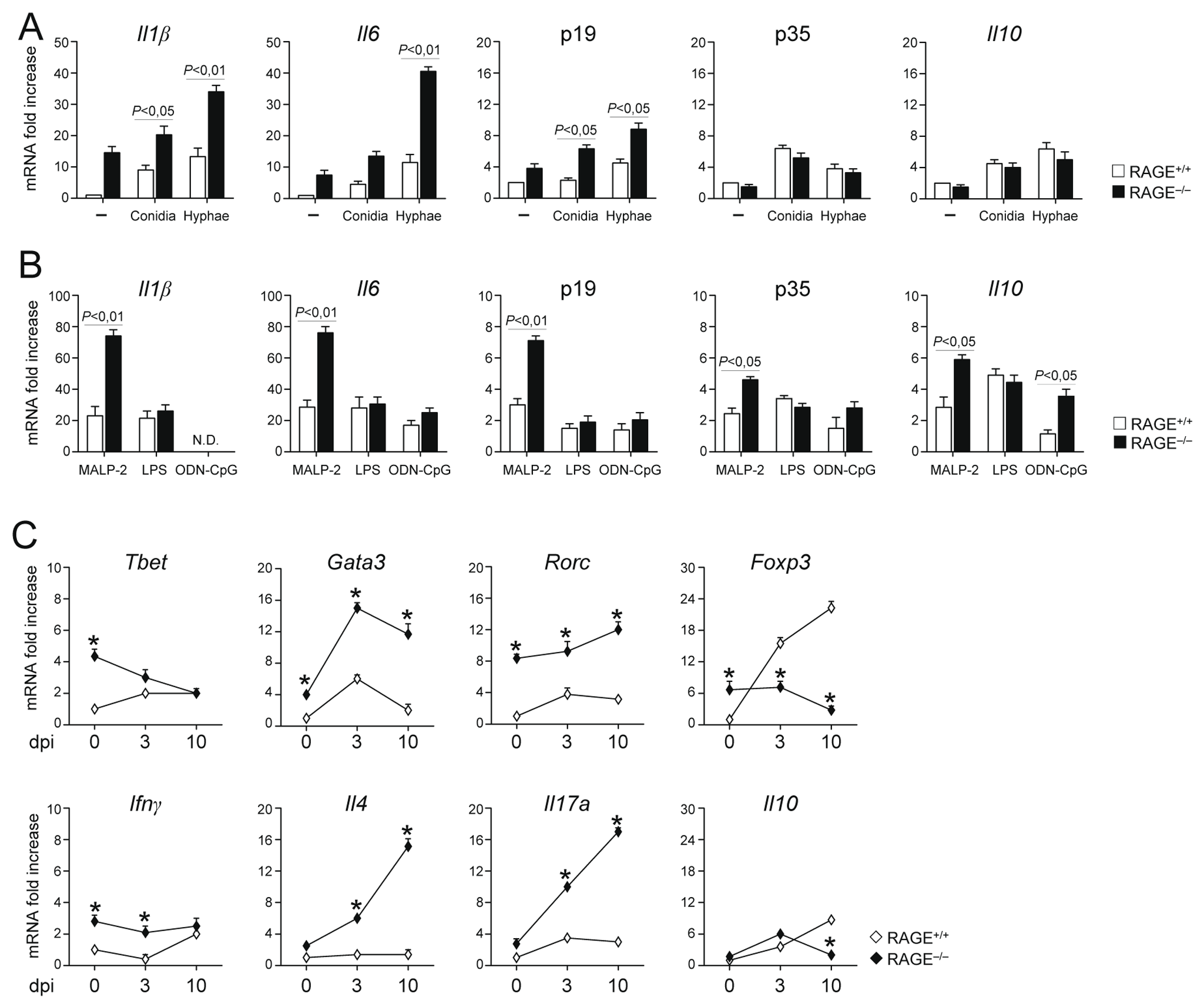

Supplement: Figure S2 — RAGE-deficient mice develop pathogen-induced Th inflammation. (A) Purified DCs from uninfected mice were exposed to live resting conidia or hyphae as described for 18 h before real time RT-PCR. (B) Cytokine gene-expression by real-time RT-PCR in DCs from RAGE KO or WT uninfected mice exposed to MALP-2, LPS or ODN-CpG for 18 h. (C) Freshly isolated CD4+T cells from TLN were assessed for transcription factor expression by RT-PCR. P, KO vs WT mice. P, KO vs WT mice. Data are pooled from 4 experiments or representative of 2 experiments (for histology). Representative of 2 experiments. P, KO vs WT DCs. N.D., not determined. (0.57 MB TIF) [file ppat.1001315.s002.tif]

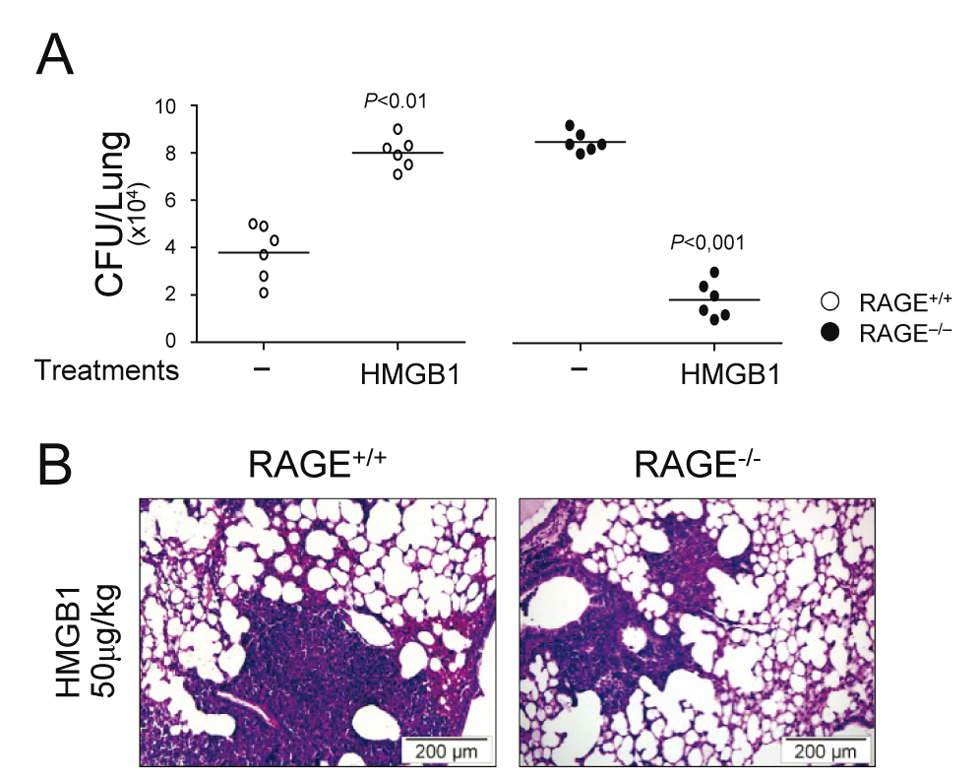

Supplement: Figure S3 — Effects of HMGB1 administration in mice with aspergillosis. Fungal growth (CFU±SE) (A) and lung histology (PAS staining) (B) in C57BL6 or RAGE KO mice infected with Aspergillus live conidia intranasally and treated intraperitoneally for 3 consecutive days with 50 µg/kg HMGB1. P, treated vs untreated (-) mice. Representative of 3 experiments. *P<0.05, treated vs untreated (-) cells. (0.65 MB TIF) [file ppat.1001315.s003.tif]

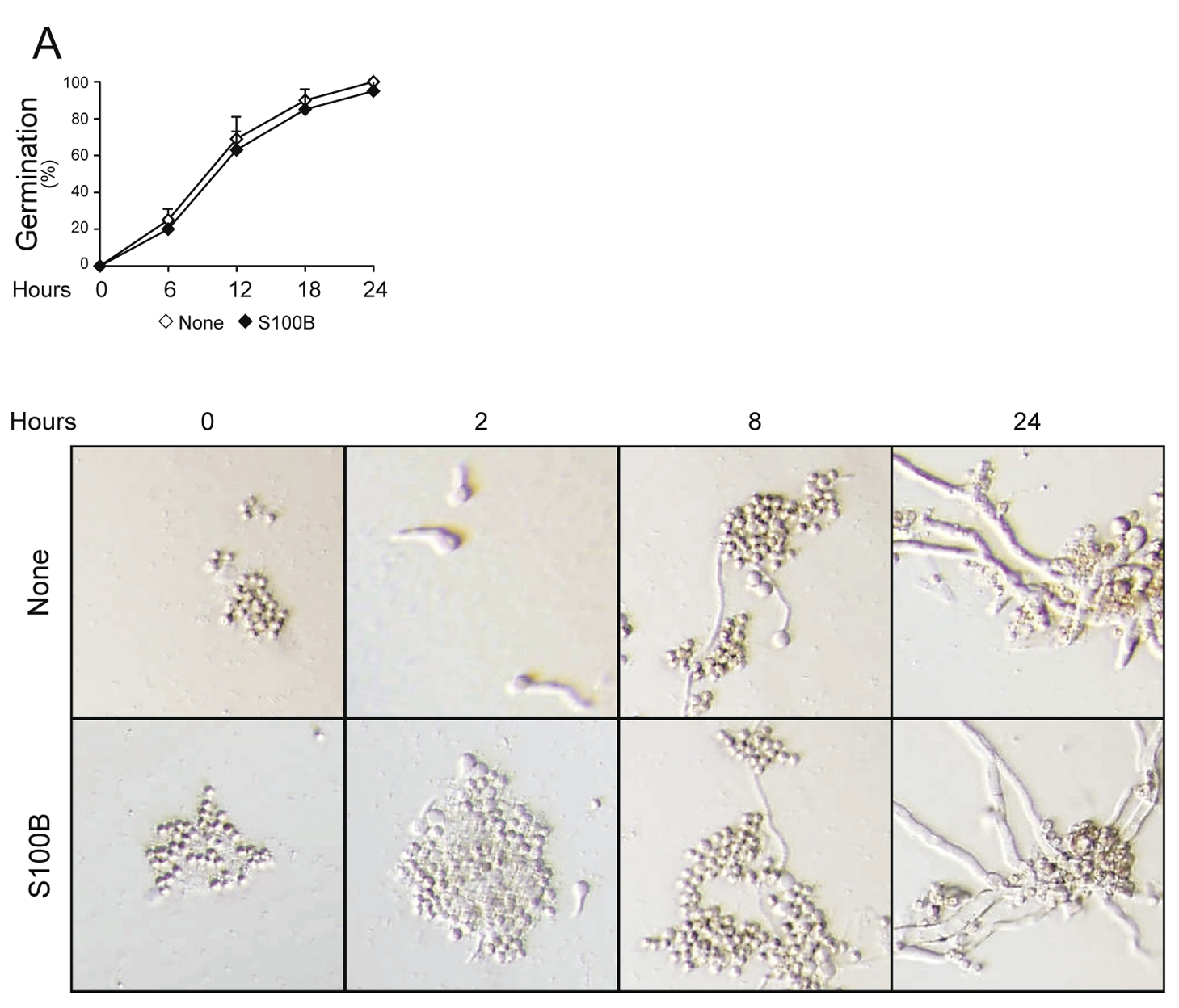

Supplement: Figure S4 — Effect of S100B on A. fumigatus morphology and germination. Germination refers to the percentages (mean ± SE) of germinating cells over a total of 400 cells counted. Magnification x 40. Shown are the pooled results from 2 experiments. Photographs were taken using a high Resolution Microscopy Color Camera AxioCam, using the AxioVision Software Rel. 3.1 (Carl Zeiss S.p.A., Milano, Italy). (1.25 MB TIF) [file ppat.1001315.s004.tif]
