# Supplementary material for: The Danger Signal S100B Integrates Pathogen– and Danger–Sensing Pathways to Restrain Inflammation
Source: PLoS Pathog. 2011 Mar 10;7(3):e1001315. doi: 10.1371/journal.ppat.1001315 (PMC3053348; doi:10.1371/journal.ppat.1001315)
Supplement: Text S1 — Supplemental methods referred to Supplemental Figures S1, S3, S4. (0.04 MB DOC) [file ppat.1001315.s005.doc]

**Text S1**

**Cell separation and cultures.** Lung CD11c^+^ DCs (between 5 to 7% positive for CD8α and between 30 to 35% positive for Gr–1) were isolated by magnetic cell sorting with MicroBeads (Miltenyi Biotec, Germany) and exposed to fungi for 18 h as described [30]. CD4^+^ cells were separated by magnetic cell sorting from thoracic lymph nodes as described [30] and freshly used for RT–PCR. DCs were exposure to vital *Aspergillus* conidia or hyphae, as described [25], 5 μg/ml MALP–2, 10 μg/ml ultrapure LPS from *Salmonella minnesota* Re 595 (all from Sigma Aldrich), and 10 μg/ml ODN–CpG [24] for 24 h before RT-PCR assay. Cultures were done in the presence of 2% FBS. Control cells were treated with PBS, DMSO or control antibody.

**HMGB1 treatment in vivo**. Mice were intranasally infected with viable *Aspergillus* conidia, treated daily i.p. for 3 consecutive days starting the day of the infection with 50 μg/kg HMGB1(Sigma Chemical Co) and monitored for fungal growth (CFU/lung, mean ± SE) and lung histology (PAS staining). Control received PBS or isotype controls (Sigma–Aldrich).

**Treatment of *A. fumigatus* with S100B.** *Aspergillus* conidia were grown in Sabouraud dextrose broth at 22°C (room temperature) for 48 h, washed in PBS and resuspended (10^5^ cells/ml) in RPMI 1640 containing from 5 to 500 nM S100B for 24 h at 37°C in 5% CO_2_. At different time points, fungal morphology was visualized by light microscopy. Results refer to 500 nM S100B. Photographs were taken using a high Resolution Microscopy Color Camera AxioCam, using the AxioVision Software Rel. 3.1 (Carl Zeiss S.p.A., Milano, Italy).
